# The commensal *Escherichia coli* CEC15 reinforces intestinal defences in gnotobiotic mice and is protective in a chronic colitis mouse model

**DOI:** 10.1038/s41598-019-47611-9

**Published:** 2019-08-07

**Authors:** Unai Escribano-Vazquez, Sophie Verstraeten, Rebeca Martin, Florian Chain, Philippe Langella, Muriel Thomas, Claire Cherbuy

**Affiliations:** Micalis Institute, INRA, AgroParisTech, Université Paris-Saclay, 78350 Jouy-en-Josas, France

**Keywords:** Physiology, Gastroenterology

## Abstract

*Escherichia coli* is a regular inhabitant of the gut microbiota throughout life. However, its role in gut health is controversial. Here, we investigated the relationship between the commensal *E*. *coli* strain CEC15 (CEC), which we previously isolated, and the intestine in homeostatic and disease-prone settings. The impact of CEC was compared to that of the probiotic *E. coli* Nissle 1917 (Nissle) strain. The expression of ileal and colonic genes that play a key role in intestinal homeostasis was higher in CEC- and Nissle-mono-associated wild-type mice than in germfree mice. This included genes involved in the turnover of reactive oxygen species, antimicrobial peptide synthesis, and immune responses. The impact of CEC and Nissle on such gene expression was stronger in a disease-prone setting, *i*.*e*. in gnotobiotic IL10-deficient mice. In a chronic colitis model, CEC more strongly decreased signs of colitis severity (myeloperoxidase activity and CD3^+^ immune-cell infiltration) than Nissle. Thus, our study shows that CEC and Nissle contribute to increased expression of genes involved in the maintenance of gut homeostasis in homeostatic and inflammatory settings. We show that these *E. coli* strains, in particular CEC, can have a beneficial effect in a chronic colitis mouse model.

## Introduction

*Escherichia coli*, a member of the *Proteobacteria*, *is* a Gram-negative facultative anaerobe with one of the most diverse lifestyles of all microbes and includes commensal, probiotic, and highly pathogenic strains^1^. This high phenotypic diversity is mirrored by the high genomic plasticity of *E*. *coli*, enriched by the acquisition of numerous mobile genetic elements.

The primary habitat of *E*. *coli* is the intestine, where it resides as a common and widespread inhabitant of the gut microbiota^2^. Upon birth, *E*. *coli* massively colonizes the gut after fetal exposure to maternal fecal matter and is predominant in the newborn gut microbiota^3^. Exposition to *E*. *coli* may even start *in utero*, as it is one of the most abundant microbes found in the maternal placental microbiota^4^. These early colonizers can change the structure and function of the intestinal epithelial in ways that may be critical for healthy microbiota development^5^ or provide a strong stimulus for B cell-maturation^6^, therefore contributing to intestinal homeostasis and immune maturation. In adulthood, *E*. *coli* remains a major member of the aero-tolerant fraction of the gut microbiota, although outnumbered by anaerobic bacteria, and stabilizes at approximately 10^7^–10^8^ CFU/g of fecal content^7^. Commensal *E*. *coli* resides on the mucus layer, for which its metabolism has adapted to use as a source of essential nutrients^8^, near the intestinal mucosa, due to the radial oxygen gradient^9^. Thus, this bacterial species, with its own nutritional requirements and metabolic capacity distinct from that of anaerobic bacteria, may play a specific role in the crosstalk between commensal bacteria and the host.

Several studies have assigned indigenous *E*. *coli* as pathobiont, a term that describes commensal microbes that induce disease only in certain genetic or environmental contexts^10^. Indeed, the disruption of the microbiota composition (or dysbiosis) that accompanies several human diseases is characterized by the expansion of *Enterobacteriaceae*, including *E*. *coli*. This has been shown for Crohn’s disease (CD)^11^, with a high prevalence of pro-inflammatory adherent-invasive *E*. *coli* (AIEC)^12^, colorectal cancer^13^, and, to a lesser extent, ulcerative colitis (UC)^14^. Similarly, the abundance of intestinal *Escherichia* also increases in several mouse models of inflammatory bowel disease (IBD)^15,16^. A more direct effect of certain indigenous strains of *E*. *coli* on intestinal inflammation has been demonstrated in gnotobiotic mouse models with a predisposition to inflammation. Thus, mono-association of mice genetically prone to inflammation with *E*. *coli* strains, originally isolated from mice gut microbiota, results in intestinal inflammation^17–19^.

However, the role of indigenous *E*. *coli* toward gut health is far from clear and requires further investigation. Indeed, another commensal *E*. *coli* isolate failed to induce disease in antibiotic-pretreated IBD-susceptible mice, despite robust colonization^20^. The inflammation-prone HLA B27 transgenic rat model responds only very moderately to *E*. *coli* strains isolated from CD patients, whereas other bacteria induce severe colitis^21^. Studies in a mono-associated IL2^−/−^ mice model report divergent effects of indigenous *E*. *coli* strains in the induction of colitis^19^. Furthermore, *E*. *coli* strains originally isolated from human gut microbiota are the basis of at least two commercially available probiotic products, commercialized under the names Mutaflor and Symbioflor2. The *E*. *coli* Nissle 1917 (Nissle) strain, the active component of Mutaflor, is one of the most thoroughly investigated and documented probiotics^22^. Clinical trials have shown a beneficial effect of Nissle for the maintenance of remission in UC, similar to that of mesalazine^23^.

We previously isolated a primo-colonizing *E*. *coli* strain called CEC15 (CEC) from freshly pooled fecal samples of 15-day-old suckling rodents as a major representative of this environment. We reported that this *E*. *coli* strain elicits sequential remodelling of the colonic epithelium in gnotobiotic rodent models, affecting different arms of the intestinal epithelial defences required to achieve a microbiota-accommodating homeostasis^5^.

Here, we aimed (1) to gain further insight into the relationship between CEC and host intestinal health and (2) to compare the intestinal host response to CEC to that of the probiotic *E*. *coli* Nissle. We explored the ileal and colonic host response to these two strains under three different conditions: (*i*) disease-free mono-associated wild-type (WT) mice, (*ii*) mono-associated IL10 deficient (IL10^−/−^) mice, predisposed to intestinal inflammation; (*iii*) conventional IL10^−/−^ mice exposed to inflammatory challenge as a chronic colitis model.

In summary, our data show that the host response to commensal strains of *E*. *coli* mobilized key genes that are involved in sustaining the symbiotic relationship with the gut microbiota. We further demonstrate that the host can over-mobilize its defence mechanisms when mono-associated with commensal *E*. *coli* in a setting in which the animals are predisposed to inflammation. Finally, we found that the impact of CEC is beneficial to the host in a chronic colitis IL10^−/−^ mouse model.

## Methods

### Ethics approval

All procedures involving animal experimentation were carried out according to the European guidelines for the care and use of laboratory animals under the authority of a license issued by the French Veterinary Services (authorization number 78–122 specific to CC) and were approved by the French “Ministère de l’Enseignement Supérieur et de la Recherche” (authorization number APAFIS#3441-2016010614307552). Experiments involving gnotobiotic WT or IL10^−/−^ mice were performed at the Anaxem facility of the MICALIS Institute (INRA, Jouy-en-Josas, France), which is accredited by the French “Direction Départementale de la Protection des Populations (DDPP78)”, accreditation number A78-322-6. Experiments involving conventional (CV) WT or IL10^−/−^ mice were performed at the IERP facility (INRA, Jouy-en-Josas, France), which is accredited by the French “Direction Départementale de la Protection des Populations (DDPP78)”, accreditation number DDPP-VET-13-0124. GF WT mice and GF IL10^−/−^ mice (generated from the CV IL10^−/−^ mice (B6.129P2-Il10tm1Cgn/J; see below) using the standard procedure of cesarean delivery), were purchased from the GF rodent breeding facilities of the CNRS-TAAM (transgenesis, archiving and animal models) center (Orléans, France). They were delivered to Anaxem under sterile conditions and immediately transferred to the experimental isolator. After reception, GF mice were left undisturbed for eight days before starting the experiment. Conventional WT and IL10^−/−^ (B6.129P2-Il10tm1Cgn/J)^24^ mice were born and bred at the IERP under standard conditions. The absence of *IL10* gene expression was verified in IL10^−/−^ GF and IL10^−/−^ CV mice. All WT GF and CV mice used in experiments were C57BL/6. The genetic background of IL10^−/−^ mice is a mix of C57BL/6 and 129/Ola. All animals used in this study were males.

### Gnotobiotic mice

Six groups of gnotobiotic mice were studied (Supplementary Fig. 1A,B): either GF WT (GF^WT^), or IL10^−/−^ (GF^IL10^); two groups of mono-colonized WT mice inoculated with either *E*. *coli* CEC or *E*. *coli* Nissle (CEC^WT^ and Nissle^WT^ groups, respectively) and two groups of mono-associated IL10^−/−^ mice inoculated with either *E*. *coli* CEC or *E*. *coli* Nissle (CEC^IL10^ and Nissle^IL10^ groups, respectively). The absence of microbes was verified in GF mice by microscopic observation of fresh feces and culturing of fecal material on various bacterial culture media. All mice were maintained in Trexler type isolators and received the same standard diet (*ad libitum*, R03-40 SAFE sterilized by gamma irradiation at 45 kGy). CEC and Nissle *inocula* were prepared from fresh overnight cultures. Bacterial pellets were obtained by centrifugation (4 °C; 20 min; 4,700 × g), re-suspended in sterile PBS, immediately introduced into the isolator and 100 µL (containing 10^8^ bacteria) used to inoculate GF mice. All mice were 8 to 10 weeks old at the time of inoculation and were sacrificed 21 days post-inoculation. Each experiment was carried out independently at least three times.

### Experimental colitis in CV IL10^−/−^ mice

Experimental chronic colitis was induced in six-to-eight-week-old CV IL10^−/−^ mice by rectal injection with dinitrobenzene sulfonic acid (DNBS) (Supplementary Fig. 1C). The protocol was carried out according to^25^, except for the dose of DNBS, which was lower in our study. Prior to DNBS administration, mice were anesthetized with Xelamine/Ketamine and a 10 cm long piece of PE-90 tubing was attached to a syringe and inserted 3.5 cm into the colon. On the first day of the protocol, mice received one rectal dose of 150 mg/kg DNBS (ICN Biomedical Inc., Santa Ana, CA) in 30% ethanol. All mice received a subcutaneous injection of 1 ml saline solution (0.9% NaCl) for three days to prevent dehydration. Mice were allowed to recover for 21 days and then received a second DNBS injection at day 21, reactivating inflammation. Ten days before sacrifice, DNBS treated CVIL10^−/−^ mice were given a dose of 1 × 10^9^ CFU of CEC (DNBS^IL10^ + CEC) or Nissle (DNBS^IL10^ + Nissle) daily by oral gavage, or the same volume of PBS as a positive control for the disease (DNBS^IL10^) (Supplementary Fig. 1C). Mice were sacrificed 24 days after the first DNBS injection, *i*.*e*. three days after the second. Run in parallel, CV IL10^/−^ mice received PBS instead of DNBS and were supplemented daily with PBS (Control), as a negative control group.

### Sample collection

The day of sample collection, at 9:00 AM, mice were anesthetized with isoflurane, killed by cervical dislocation, and the ileum and colon promptly removed. An unflushed section of ileum and colon was kept and fixed in CARNOY (four weeks at 4 °C) for mucus layer thickness measurements as in^5^. The remainder of the intestinal tissues was quickly washed with PBS and immediately used either for *ex vivo* permeability measurements in an Ussing chamber, or fixed in 4% paraformaldehyde (PFA; 6 h at room temperature) for further histological analyses, or frozen at −80 °C for RNA extraction. Cecal contents were recovered and frozen at −80 °C before DNA extraction for 16S rRNA gene sequencing.

### Histology and Immunostaining

CARNOY- and PFA-fixed ileum and colon were dehydrated and embedded in paraffin according to a standard protocol^26^. Staining was performed with Dako reagents according to the recommendations of the manufacturer. For PFA fixed samples, antigen retrieval was performed by boiling the slides for 40 min in 10 mM tri-sodium citrate pH 6.0. The primary antibodies used and corresponding dilutions were: anti-cadherin1 (Cdh-1, Invitrogen; 1/50), anti-CD3 (Abcam, 1/500), anti-mucin-2 (Muc2, Santa Cruz; 1/500), anti-mucin-13 (Muc13, Santa Cruz; 1/500), and anti-Ki67 (Dako; 1/50). Negative controls were performed by omitting the primary antibody from the reaction. For each section, Ki67+ cells were counted for 10 crypts and the results expressed as the percentage of total crypt cells. CD3^+^ cells were counted by microscopic field (x200). For mucus thickness analyses, CARNOY-fixed cuts were stained with the Muc-2 antibody. Tissues were observed with a 3DHistech Panoramic Scan and signal quantification was performed using Panoramic Viewer® or ImageJ software.

### RNA extraction and cDNA preparation for real-time RT-PCR experiments

RNA isolation was carried out using the Ambion mirVana Kit (Life Technologies) according to the manufacturer’s recommendations. The RNA quality of all samples was checked using an Agilent 2100 Bioanalyzer (Agilent Technologies, Santa Clara, CA, USA) with an average RNA integrity number superior to 9 on a scale of 0 to 10. Complementary DNA (cDNA) was prepared using the RT RevertAid H Minus first Strand cDNA Synthesis Kit (Life Technologies).

### Gene expression profiling using the TaqMan OpenArray System

For studies in gnotobiotic WT or IL10^−/−^ mice, gene expression analysis was performed with a customized TaqMan OpenArray Real-time PCR System (Life Technologies). We designed two TaqMan OpenArrays, one dedicated to the ileum, the other to the colon, following a thorough review of the literature and searching the NCBI public repository Gene Expression Omnibus. For each TaqMan OpenArray, 220 genes were selected, plus four endogenous control genes (*actb*, *gapdh*, *ubc*, and *tpb*). The candidate genes selected for this study survey various functions of ileal and colonic cells, mainly involved in mucosal defence: immunological responses, intestinal barrier, oxidative stress, anti-microbial peptides, cellular signalling, regulation of cell proliferation and differentiation, detoxification, DNA-damage detection, growth factors, inflammasome, inflammation pathway, lipid synthesis and metabolism, pattern recognition, and solute transport (Supplementary Table 1–2, for the list of genes included in the ileum and colon array cards, respectively). For studies in DNBS treated Il10^−/−^ mice, gene expression profiling was performed using the TaqMan OpenArray Mouse Inflammation Panel plate (Life Technologies) designed by the manufacturer, that consists of 632 gene targets selected for their involvement in inflammatory responses. The cDNA (10 µl) was mixed with the TaqMan OpenArray Real-Time PCR Master Mix and loaded onto the cards using the AccuFill™ System. The cards were cycled in an OpenArray NT Cycler System (Applied Biosystems) at the integrative microgenomic platform (@BRIDGe, INRA, Jouy-en-Josas) following the manufacturer’s protocol. The same sample was systematically loaded in each TaqMan OpenArray and used to check the reproducibility between the plates. Data were extracted using OpenArray Real-Time qPCR Analysis software (Applied Biosystems). The fold-change in gene expression (Rq or relative quantification) was calculated using the comparative 2^−ΔΔCq^ method with global normalization of all gene expression data using GenEx software (Multid Analyses)^27^. The GF group was used as a calibrator for the gnotobiotic experiments and the Control group for gene expression profiling of the DNBS treated CV IL10^−/−^ mice.

### Principal component analysis, heatmap representation, and statistical analysis of the TaqMan OpenArray data

The R statistics environment was used for data analyses. Data obtained from TaqMan OpenArray experiments are represented either by Principal Component Analysis (PCA) plots or heatmaps. Rq values were used to build the PCA plots and heatmaps. Prior to PCA, we filtered the data using a one-way permutation test (oneway_test, R package “coin”) to remove genes from the datasets for which the variation in expression was less informative (threshold set to p < 0.01). PCA was then carried out on the filtered dataset using the R packages FactoExtra and FactoMineR. The number of genes included in the PCA analysis is specified in the figure legend for each comparison. Heatmaps were generated for genes showing a significant difference between the mono-associated mice and the GF group (p < 0.05; Man-Whitney test). Clustering on the gene expression profile was applied to the genes in the heatmap.

### Single real-time quantitative PCR analyses of gene expression

Single real-time quantitative PCR assays were used to confirm the results obtained on the TaqMan Open Arrays system using the corresponding TaqMan assay. All gene expression results are expressed with the 2^−∆∆Ct^ method (Rq), using *gapdh* as the housekeeping gene and values from the GF (gnotobiotic experiments) or Control (Chronic colitis experiments) mice as calibrators^27^.

### *Ex vivo* intestinal permeability measurements

After removal, segments of the ileum and colon were immediately mounted in Ussing chambers, as previously described^5^. Para-cellular permeability was further assessed by measuring mucosal-to-serosal flux of 4 kDa non metabolizable fluorescein isothiocyanate-labeled dextran (FD4) or 0.4 kDa FITC-sulfonic acid (FSA) for 90 min. Molecules were added to the mucosal side of the chamber at a final concentration of 0.4 mg/mL and the fluorescence intensity determined at the serosal side. Trans-epithelial conductance was measured by clamping the voltage and recording the change in the short-circuit current (Isc). At the end of the experiment, tissues were challenged with the cholinergic analog carbachol (CCh) on the serosal side (100 mM) and the ΔIsc was recorded to check the viability of the tissue.

### Myeloperoxidase activity

Myeloperoxidase (MPO) activity was assayed according to^25^. MPO activity is expressed as the units per milligram of total protein. Lowry’s method was used for protein quantification.

### 16S rRNA gene sequencing and analysis

Cecal DNA of CV IL10^−/−^ mice was extracted as described in^28^ and the V3-V4 hyper-variable region of the 16S rRNA gene amplified with the primers F343 (CTTTCCCTACACGACGCTCTTCCGATCTTACGGRAGGCAGCAG) and R784 (GGAGTTCAGACGTGTGCTCTTCCGATCTTACCAGGGTATCTAATCCT). The PCR amplicons were sent to the GeT-PlaGe platform (INRA, Toulouse) for sequencing using Illumina Miseq technology. Single multiplexing was performed and the multiplexes purified and loaded onto the Illumina MiSeq cartridge according to the manufacturer’s instructions. Raw sequences were analyzed using the FROGS pipeline to obtain the OTU (operational taxonomic units or phylotypes) abundance table, phylogenetic tree, and taxonomic table using the default parameters^29^. Statistical analyses of the 16S rRNA sequences were performed from the FROGS-generated outputs using R and environment version 3.2.3. Calculations of within-community diversity (α-diversity), between-community diversity (β-diversity), and relative abundance taxonomic summaries were performed using the add-on package “Phyloseq”.

### Statistical analyses

R 3.3.1 and GraphPad 5 software were used to produce graphs and for statistical analyses. Data are expressed as the mean ± standard deviation in scatter plots. Differentially expressed genes from OpenArray data were obtained with non-parametric multiple comparison tests using false discovery rate (FDR) corrected p-values (the threshold for significance of differential expression was set to p < 0.05). Comparisons of other quantitative variables were performed using the non-parametric Mann-Whitney test (p < 0.05 was considered significant).

## Results

### Ileal and colonic gene expression profiles of CEC or Nissle mono-associated mice differ from those of GF mice

**Figure 1:**
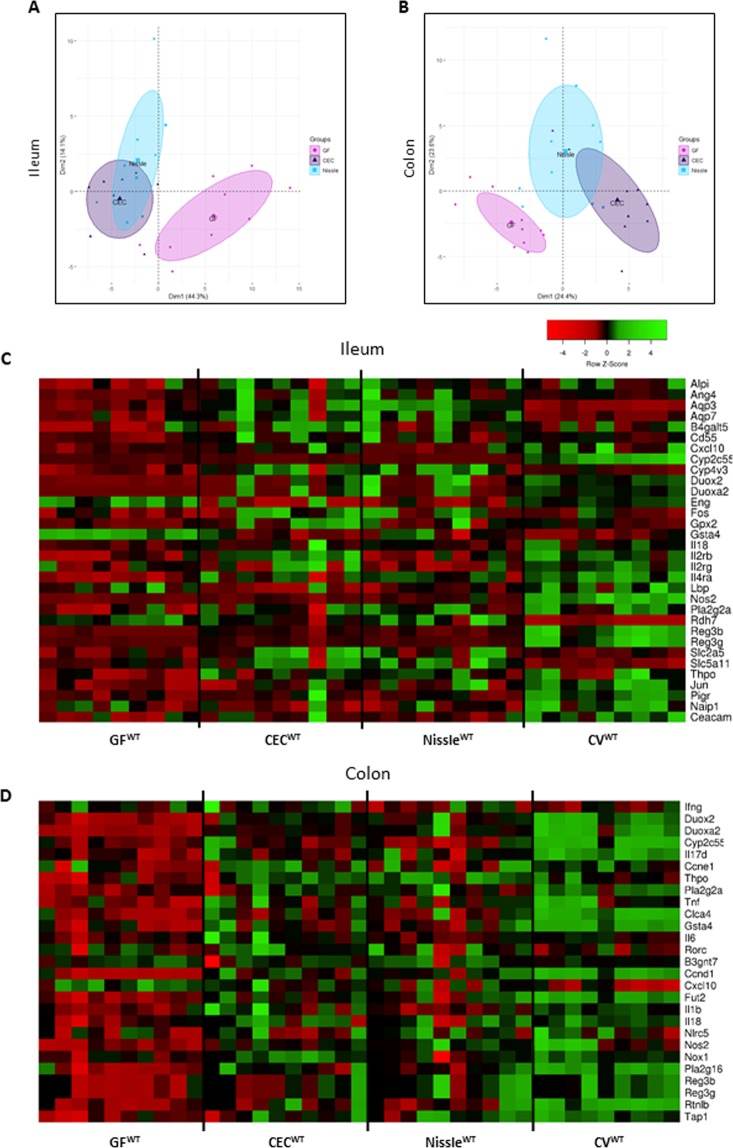
Intestinal gene expression profiles differ between GF and *E*. *coli* mono-associated WT mice. Germ-free wild-type mice (GF^WT^) were inoculated with the commensal CEC (CEC^WT^) or probiotic Nissle 1917 (Nissle^WT^) *E*. *coli* strain. Mono-colonized mice, CEC^WT^ and Nissle^WT^, were sacrificed 21 days post-inoculation and the ileal and colonic gene expression profiles analyzed by high-throughput qPCR using TaqMan OpenArrays. (**A**) and (**B**) principal component analysis (PCA) plots of gene expression data in the ileum and colon, respectively. After pre-filtering the dataset, 64 genes were included for the PCA, both for the ileum and colon; n = 9–10 mice per group. (**C**) and (**D**) Gene-expression heatmaps for the ileum and colon, respectively. Genes that are shown are significantly differentially expressed relative to GF^WT^.

We carried out gene expression profiling by high-throughput open-array qPCR of the ileum and colon of CEC^WT^, Nissle^WT^, and GF^WT^ mice (Fig. 1) using two customized gene-expression array plates. One was designed for the ileum and the other the colon, with a selection of candidate genes mainly involved in mucosal defence of the intestine (Supplementary Tables 1, 2). PCA revealed a clear separation of the gene expression profile between mono-associated and GF^WT^ mice, both in the ileum (Fig. 1A) and colon (Fig. 1B). Accordingly, in the ileum, PC1 separated the mono-associated CEC^WT^ and Nissle^WT^ groups from the GF^WT^ group, with both mono-associated groups overlapping. In the colon, PC1 also separated the gene expression profiles of the GF^WT^ and mono-associated groups. The mono-associated CEC and Nissle groups tended to slightly diverge along PC2, revealing a stronger effect of the CEC strain on the colonic gene expression profile. We also included CV^WT^ mice for comparison with the CEC^WT^, Nissle^WT^, and GF^WT^ mice (Supplementary Fig. 2). As expected, PCA first separated the CV^WT^ from all gnotobiotic groups along PC1. In the ileum and colon, the CEC^WT^ and Nissle^WT^ groups clustered together and were distinct from the GF^WT^ and CV^WT^ groups (Supplementary Fig. 2).

We next selected a subset of genes from high-throughput qPCR data that were differentially expressed between the GF^WT^ and mono-associated groups and exhibited a Rq value > 1.5. Gene expression variation were plotted in a heatmap (Fig. 1C for the ileum and Fig. 1D for the colon). Again, there was a clear impact of the CEC and Nissle strains on the gene expression profile at both sites of the intestine (Fig. 1C,D). Differences in gene expression were confirmed in single TaqMan assays (Examples given in Supplementary Fig. 3 for the ileum and Supplementary Fig. 4 for the colon). Genes involved in several key intestinal functions in the ileum and colon of mice mono-associated with CEC or Nissle were upregulated. Indeed, genes were upregulated for: *i*) enzymes involved in ROS/RNS turnover, such as *duox2* (dual oxidase 2), *duoxa2* (dual oxidase activator 2), and *nos2* (nitric oxide synthase 2) for both the ileum and colon; *gpx2* (glutathione peroxidase 2) for the ileum only; and *nox1* (NADPH oxidase 1) for the colon only; *ii*) antimicrobial peptide production and barrier functions: *reg3-γ*, *reg3-β* (regenerating islet-derived-*γ and -β*) and *pla2g2a* (secretory phospholipase A group IIA) for both the ileum and colon, *ang4* (angiogenin-4) for the ileum only, and *fut2* (α(1,2)-fucosyltransferase) for the colon only; and *iii*) factors involved in the immune response, such as *IL18* (interleukin-18), *cxcl10* (C-X-C motif chemokine ligand 10), *tnf*α (tumor necrosis factor α) and *tap1* (transporter associated with antigen processing) for both the ileum and colon; *IL2rb*, *IL4ra* and *IL2rg* for the ileum only; and *IL1β*, *IL6*, *IL17d*, *thpo* (thrombopoietin), and *ifnγ* (interferon-γ) for the colon only (Fig. 1C,D) and (Supplementary Figs 3 and 4). Furthermore, CEC or Nissle modulated genes involved in the transport function of the intestine, such as the fructose transporter *slc2a5* (solute carrier family 2 member 5; previously called *glut5*) and members of the aquaporin water channel family *aqp*3 and *aqp7*, which were dramatically upregulated in the ileum of CEC^WT^ and Nissle^WT^ mice, and the gene *clca4* (Ca 2+-activated chloride channel 4), which was upregulated in the colon (Fig. 1C,D) and (Supplementary Figs 3 and 4).

### CEC and Nissle maintain intestinal barrier function and epithelial integrity in gnotobiotic WT mice

**Figure 2:**
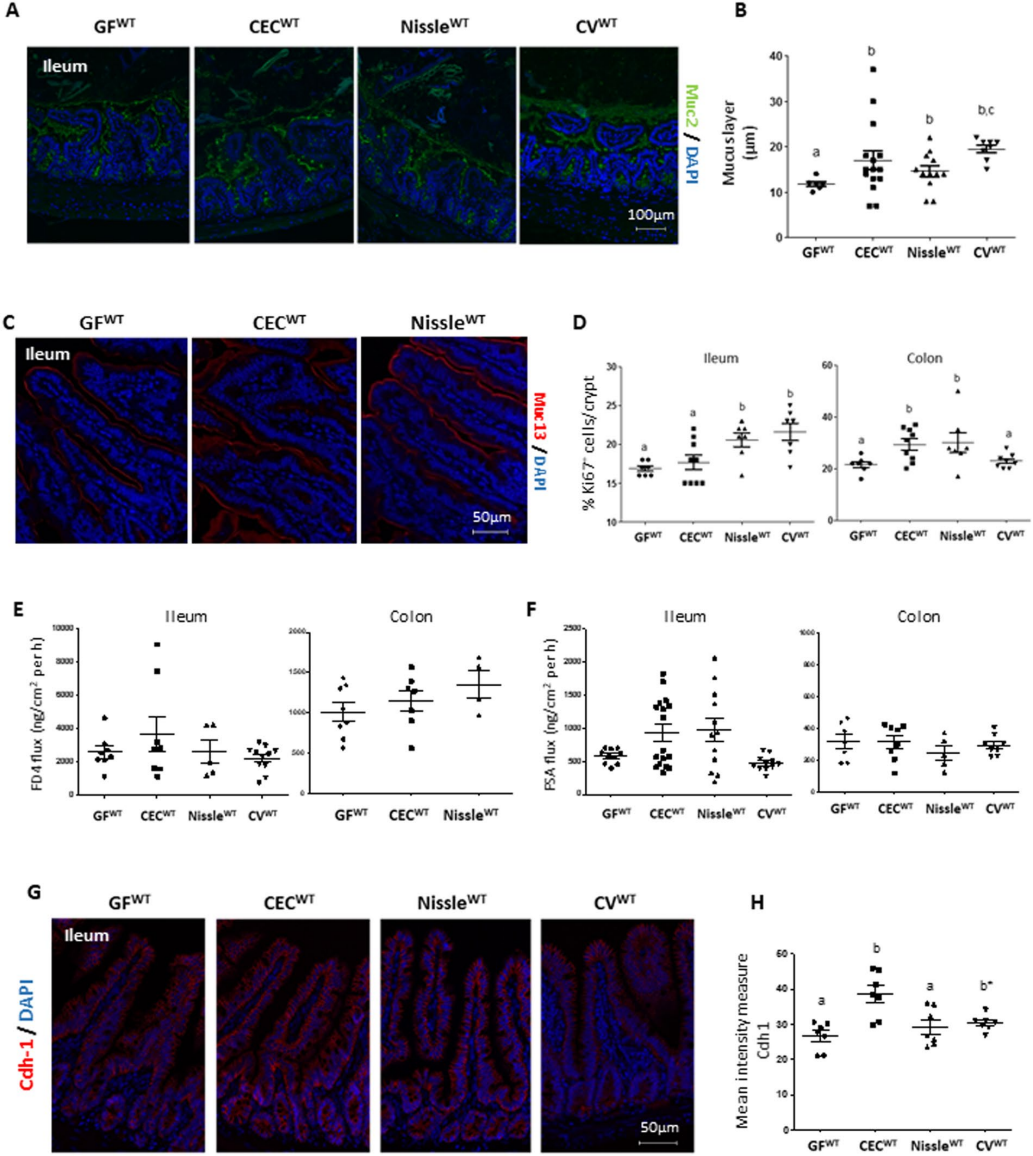
Intestinal barrier function is preserved in *E*. *coli* mono-associated WT mice. Analyses of intestinal barrier markers were carried out on germ-free wild-type mice (GF^WT^), on mice mono-colonized with the CEC (CEC^WT^) or Nissle (Nissle^WT^) strains, and on conventional WT mice (CV^WT^). (**A**) Representative images of Muc2 (green) immunostaining of CARNOY-fixed tissues. (**B**) Thickness measurement of the ileal mucus layer (green layer at the top of the epithelium), n = 7–15 mice per group. (**C**) Muc13 (red) immunostaining of PFA-fixed tissues. Nuclei are stained with DAPI (blue). (**D**) Percentage of Ki67^+^ cells per total cells per crypt for the ileum and colon, n = 7–9 mice per group. (**E**) and (**F**) Analysis of *ex vivo* para-cellular permeability using the Ussing chamber system with (**E**) FITC-dextran (4 KDa; FD4) and (**F**) FITC-sulfonic acid (400 Da; FSA) or for the ileum and colon, n = 5–17 mice per group. (**G**) Immunostaining of the adhesion protein cadherin-1 (Cdh-1) in the ileum of PFA-fixed tissues. (**H**) Mean intensity of Cdh-1 staining, n = 5–7 mice/group. All values are presented as the means ± SEM. Mean values with letter designations are significantly different (non-parametric Mann-Whitney test).

We further investigated the impact of the CEC and Nissle strains on the barrier formed by the network of intestinal mucins, as it is a key player in physico-chemical protection. We examined the thickness of the mucus layer formed by the highly glycosylated secreted mucin2 (Muc2), and the abundance of membrane-associated mucins, Muc13 and Muc4. Muc2 staining showed the ileal mucus layer to be thicker in mono-colonized CEC^WT^ and Nissle^WT^ mice than GF^WT^ mice, tending to become similar to that of CV^WT^ mice (Fig. 2A,B). Similarly, Muc13 staining in the ileum of both CEC^WT^ and Nissle^WT^ mice was stronger than that of control GF^WT^ mice (Fig. 2C), but not in the colon (Supplementary Fig. 5A). There were also no modifications in Muc4 staining for any of the groups (Supplementary Fig. 5B). We also investigated the effect of the two strains on expression of the epithelial proliferation marker Ki67 (Fig. 2D). In the Nissle^WT^ group, the percentage of Ki67^+^ cells was higher than that in the GF^WT^ group in both the ileum and colon (Fig. 2D), whereas there was a significant increase in the percentage of proliferative epithelial cells in only the colon in the CEC^WT^ group (Fig. 2D).

We then assessed the effect of *E*. *coli* strains on intestinal permeability in the ileum and colon. We monitored the para-cellular passage from the mucosal to serosal side of high- and low-molecular weight molecules, FD4 (Fig. 2E) and FSA (Fig. 2F), respectively, in an Ussing chamber. There were no differences in permeability between the groups. Among the panel of tight junction or cell adhesion proteins we tested [claudins-4,-5,-7,-8,-12,-15, -17, ZO-1 and F11r (data not shown), Cld2 and Cld3 (Supplementary Fig. 5C,D)], we only observed greater immunostaining of cadherin-1 (Cdh1) in the CEC^WT^ than the GF^WT^ mice (Fig. 2G,H). These data show that intestinal barrier function and epithelial integrity are preserved in gnotobiotic WT mice.

### Key genes involved in mucosal defense and immune response are strongly mobilized in IL10^−/−^ mono-associated mice

Gnotobiotic IL10^−/−^ mice have been previously used as a model to investigate the colitis-inducing potential of individual bacterial strains, including indigenous strains of *E*. *coli*^17,18^. The colitis that IL10^−/−^ mice develop requires microbial exposure, as GF mice are protected from disease^17,18^. We investigated the effect of CEC and Nissle in mono-associated IL10^−/−^ mice, three weeks post-colonization, a time interval previously used to observe the effect of colitogenic indigenous *E*. *coli* strains in 129S6/SvEv IL10^−/−^ mice^18^. There was no weight loss nor weight differences between GF^IL10^, CEC^IL10^, and Nissle^IL10^ mice (data not shown). There was also no histological mucosal damage to the intestines of CEC^I1L0^ or Nissle^IL10^ mice (Supplementary Fig. 6).

**Figure 3:**
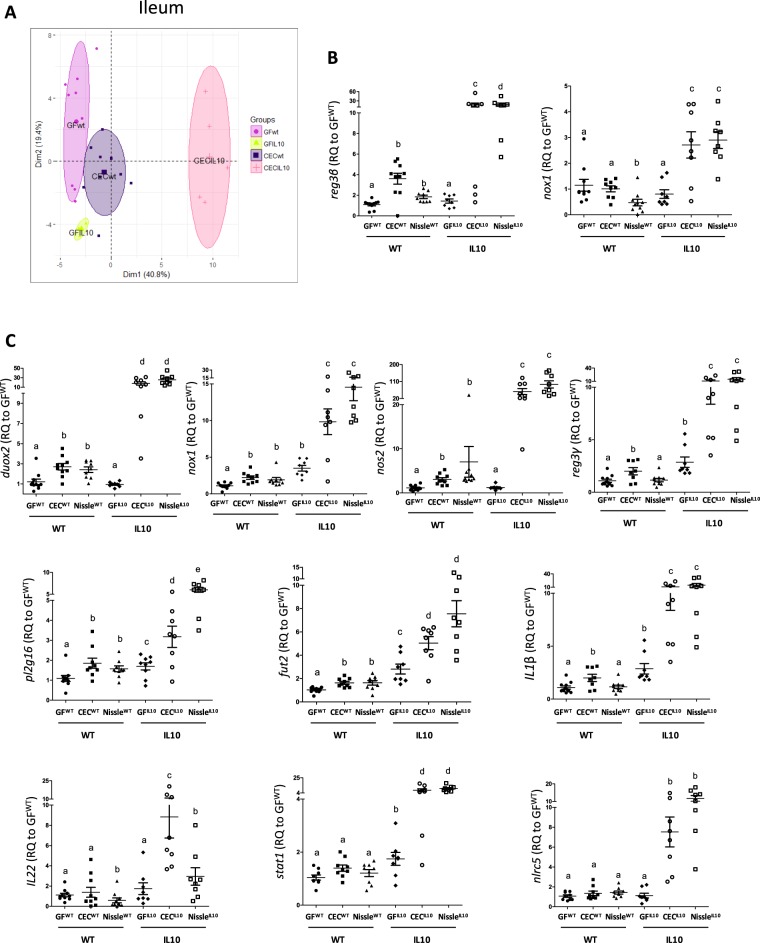
Key genes involved in mucosal defense and immune response are mobilized in IL10^/-^ mono-associated mice. Gene expression profiling of the ileal epithelium of germ-free wild-type or IL10^−/−^ mice (GF^WT^, GF^IL10^), or wild-type or IL10^−/−^ mice mono-associated with the CEC (CEC^WT^; CEC^IL10^) or Nissle (Nissle^WT^; Nissle^IL10^) strain for 21 days. (**A**) Principal component analyses (PCA) plot of gene expression data in the ileum of GF^WT^, CEC^WT^, GF^IL10^, and CEC^IL10^; 61 genes were included in the PCA; n = 4–9 mice per group. (**B**) and (**C**) Relative gene expression (Rq), measured with single TaqMan Assays, of the ileum (*reg3β* and *nox1*) and colon (*duox2*, *nox1*, *nos2*, *reg3γ*, *pl2g16*, *fut2*, *IL1β*, *IL22*, *stat1 and nlrc5*) of GF^IL10^, CEC^WT^, CEC^IL10^, Nissle^WT^, Nissle^IL10^, and CV^WT^ relative to GF^WT^, n = 8–11 mice/group. All values are presented as the means ± SEM. Mean values with letter designations are significantly different (non-parametric Mann-Whitney test).

We further investigated the effect of CEC and Nissle on the ileal and colonic gene expression profile in gnotobiotic IL10^−/−^ mice. We first randomly sampled four of the 10 individuals belonging to the GF^IL10^ and CEC^IL10^ groups to assess the ileal gene expression profile using the customized gene-expression array plates. The data obtained were then compared to those previously obtained for GF^WT^ and CEC^WT^ mice. PCA showed the CEC^IL10^ group to strongly diverge from the others along PC1 (Fig. 3A). There was a striking difference of the gene expression profile from that of the CEC^WT^ group, and the difference between the CEC^IL10^ and GF^IL10^ groups was higher than that between the CEC^WT^ and GF^WT^ groups. This initial screening suggests that the intestinal responses to *E*. *coli* of IL10^−/−^ mice are different from those of WT mice. Indeed, CEC and Nissle may have a stronger effect on the expression of ileal genes in IL10^−/−^ than WT mice, such as shown for ileal *reg3β* and *nox1* by qPCR single assays (Fig. 3B).

We investigated whether this difference in the effect of CEC and Nissle between IL10^−/−^ and WT mice also occurred in the colon by targeting key genes involved in mucosal defense using single qPCR assays (Fig. 3C). Thus, we investigated colonic expression of genes involved in the ROS/RNS turnover (*duox2*, *nox1*, *nos2*), antimicrobial peptide production (*reg3γ* and *pl2g16*), the mucosal intestinal barrier (*fut2*), immune response (*IL1β*,*IL22*), as well as *stat1*, which orchestrates antimicrobial responses and nod-like receptors 5 (*nlrc5*), an intracellular protein involved in the detection of microbes. These genes were more highly expressed in IL10^−/−^ than WT mice, when they are mono-associated with CEC or Nissle (Fig. 3C).

The differential effect of the strains on IL10^−/−^ and WT mice was not due to colonization levels, as both strains similarly colonized the WT and IL10^−/−^ mice at a level of 10^10^ bacteria/g of stool (Supplementary Fig. 7). Overall, these results show that genes involved in intestinal defense mechanisms are strongly mobilized in the presence of CEC and Nissle in IL10^−/−^ mice.

### Intestinal barrier function is not altered in Il10^−/−^ mice mono-colonized with CEC and Nissle

**Figure 4:**
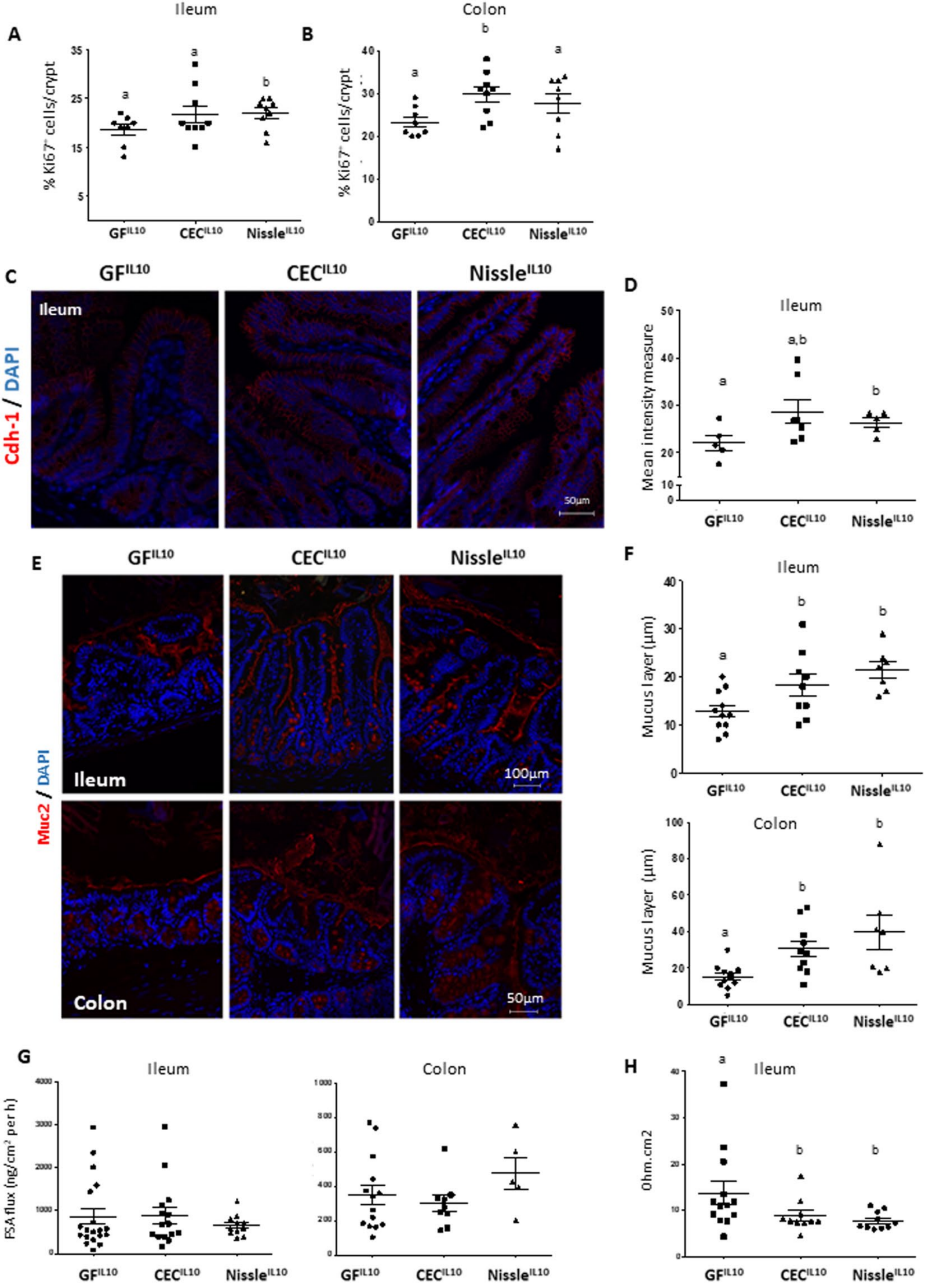
CEC15 and Nissle 1917 strengthen parameters of the intestinal barrier function in gnotobiotic Il10^−/−^ mice. (**A**,**B**) Percentage of Ki67^+^ cells per total cells per crypt of the ileum (**A**) and colon (**B**) of germ-free IL10^−/−^ mice (GF^IL10^), or IL10^−/−^ mice mono- colonized with the CEC (CEC^IL10^) or Nissle 1917 (Nissle^IL10^) strain, n = 8–9 mice/group. (**C**) immunostaining of the tight junction protein cadherin-1 (Cdh-1) of the ileum of PFA-fixed tissues. (**D**) Mean intensity of Cdh-1, n = 5–7 mice/group. (**E**) Muc2 immunostaining of the ileum and colon of GF^IL10^, CEC^IL10^ and Nissle^IL10^ mice. Tissues were fixed in CARNOY and nuclei were stained with DAPI (blue). (**F**) Quantification of mucus layer thickness (red layer on top of the epithelium) in the ileum and colon, n = 7–11 mice/group. (**G**) Analysis of *ex vivo* para-cellular permeability with FITC-sulfonic acid (400 Da; FSA) of the ileum and colon. (**H**) Conductance of the ileum, n = 10–13 mice/group. (**G**,**H**) were measured using the Ussing chamber system, n = 5–19 mice/group. All values are presented as the means ± SEM. Mean values with letter designations are significantly different (non-parametric Mann-Whitney test).

We assessed different markers to investigate whether intestinal barrier function, at the ileal and colonic level, is modified in Il10^−/−^ mice mono-colonized with CEC and Nissle: Ki67 (Fig. 4A,B), cadherin1 (cdh1), a protein involved in cell adhesion (Fig. 4C,D) and the thickness of the mucus layer (Fig. 4E,F). The number of proliferative epithelial cells in the ileum and the colon of IL10 deficient mono-colonized mice was higher than that of the GF^IL10^ group (Fig. 4A,B for the ileum and colon respectively). Ileal Cdh1 staining was greater in mono-colonized IL10 deficient mice than in GF^I1L0^ mice (Fig. 4C,D). Furthermore, the mucus layer in the ileum was thicker in the 2 mono-colonized groups than in GF^IL10^ mice, similar to our observations for WT mice (Fig. 4E,F). But in contrast to WT mice, this increase also occurred in the colon of IL10^−/−^ mice (Fig. 4E,F). Intestinal permeability was assessed *in vitro* at the level of the ileum and the colon. No difference in FSA passage among the groups was observed (Fig. 4G). In contrast, the ileal transepithelial conductance of CEC^IL10^ and Nissle^IL10^ mice was lower that than of the GF^IL10^ mice (Fig. 4H), suggesting a tendency to a lower intestinal permeability. Taken as a whole, this data reveals that CEC and Nissle do not alter intestinal permeability in a model of predisposition to inflammation. These strains can even impact positively on some markers of epithelial barrier function.

### CEC and Nissle partially reduce the severity of inflammation of DNBS-treated CV IL10^−/−^ mice

**Figure 5:**
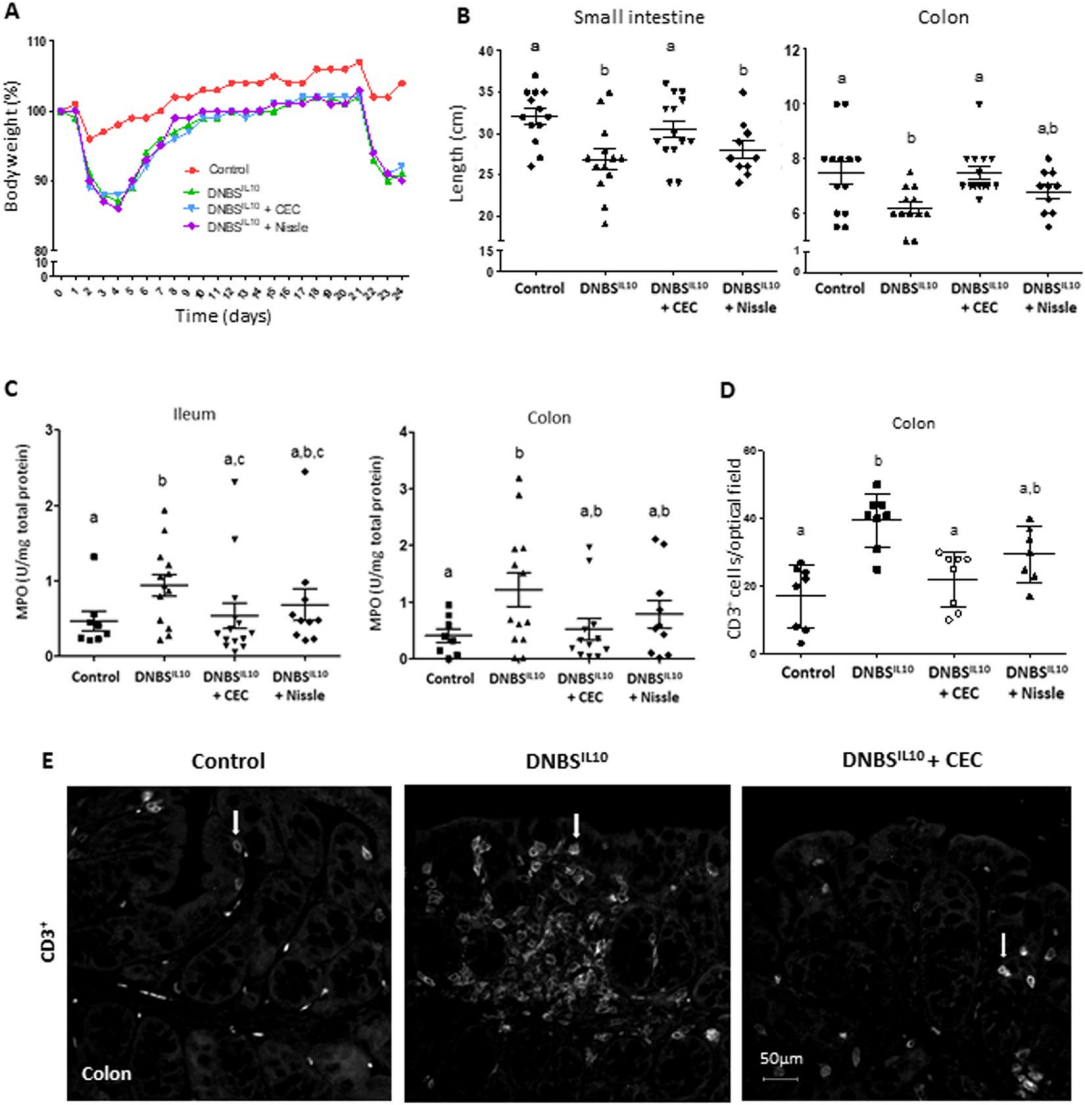
CEC15 decreases inflammation markers in DNBS-treated CV IL10^−/−^ mice. Conventional IL10^−/−^ mice treated intra-rectally with dinitrobenzene sulfonic acid (DNBS) were supplemented with CEC15 (DNBS^IL10^ + CEC) or Nissle 1917 (DNBS^IL10^ + Nissle) or PBS (DNBS^IL10^); a group of mice, that received intra-rectally PBS instead of DNBS, was used as a negative control for the disease (Control). (**A**) Mean weight evolution during the 24 days of the experiment. DNBS was injected on days 1 and 21, n = 19–26 mice/group. (**B**) Length of the small intestine and colon, n = 10–14 mice/group. (**C**) Levels of myeloperoxidase (MPO), a neutrophil infiltration marker, in the ileum and colon, n = 9–14 mice/group. (**D**) Number of CD3^+^ cell in the colonic mucosa, n = 9–12 mice/group. (**E**) Representative picture of CD3^+^ (white arrows) staining. All values are presented as the means ± SEM. Mean values with letter designations are significantly different (non-parametric Mann-Whitney test).

We next investigated the effect of CEC on inflammation using DNBS-treated CV IL10^−/−^ mice as a model of a chronic intestinal colitis. Although the Ameho score was unexpectedly close to zero (Supplementary Fig. 8), DNBS treated CV IL10^−/−^ mice exhibited several signs of inflammation that were apparent in both the ileum and colon: the mice showed a decrease in body weight (Fig. 5A), shortening of the small intestine and colon (Fig. 5B), increased myeloperoxidase (MPO) activity, a marker of neutrophil infiltration (Fig. 5C), infiltration of immune cells (CD3^+^ T lymphocytes) (Fig. 5D,E), a pro-inflammatory gene expression profile (Supplementary Fig. 9A,B).

Although CEC and Nissle were unable to induce recovery of the body weight in the model (Fig. 5A), both strains rescued the inflammation-associated reduction of ileal and colon length and MPO activity to control values (Fig. 5B,C). CEC had an additional positive effect on the number of ileal and colonic CD3^+^ T lymphocytes (Fig. 5D,E). Moreover, the permeability of the intestinal barrier did not increase after CEC treatment (Supplementary Fig. 10).

### The gene expression profile of *E*. *coli* DNBS-treated groups was skewed towards an anti-inflammatory profile, close to that of the control group

**Figure 6:**
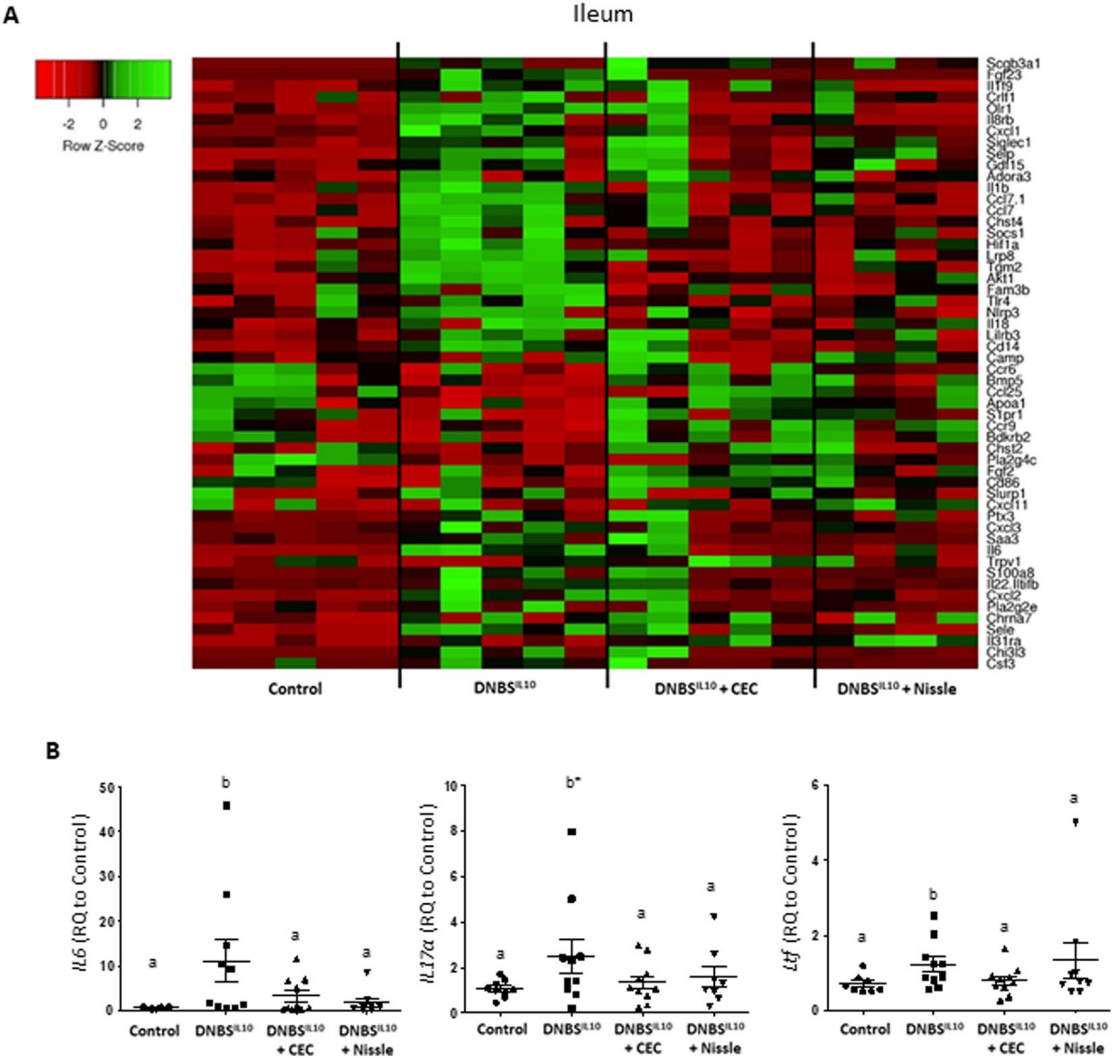
Ileal gene expression reverts towards the control profile when DNBS-treated IL10^−/−^ mice receive CEC15 or Nissle1917. Conventional IL10^−/−^ mice were treated intra-rectally with dinitrobenzene sulfonic acid (DNBS) and supplemented with CEC15 (DNBS^IL10^ + CEC), Nissle 1917 (DNBS^IL10^ + Nissle) or PBS (DNBS^IL10^), as a positive control for the disease; a group of mice, that received intra-rectally PBS instead of DNBS, was used as a negative control for the disease (Control). Gene expression profiling of the ileal mucosa was performed using the TaqMan Open Array system Mouse Inflammatory Panel. (**A**) Heatmap of the relative gene expression (Rq) to that of the Control group of the main modified genes of the ileum (**B**) Relative expression (Rq), measured with single TaqMan Assays of *IL6*, *IL17α* and *ltf* (lactotransferrin) in the ileum;n = 8–10 mice/group. All values are presented as the means ± SEM. Mean values with letter designations are significantly different (non-parametric Mann-Whitney test).

**Figure 7:**
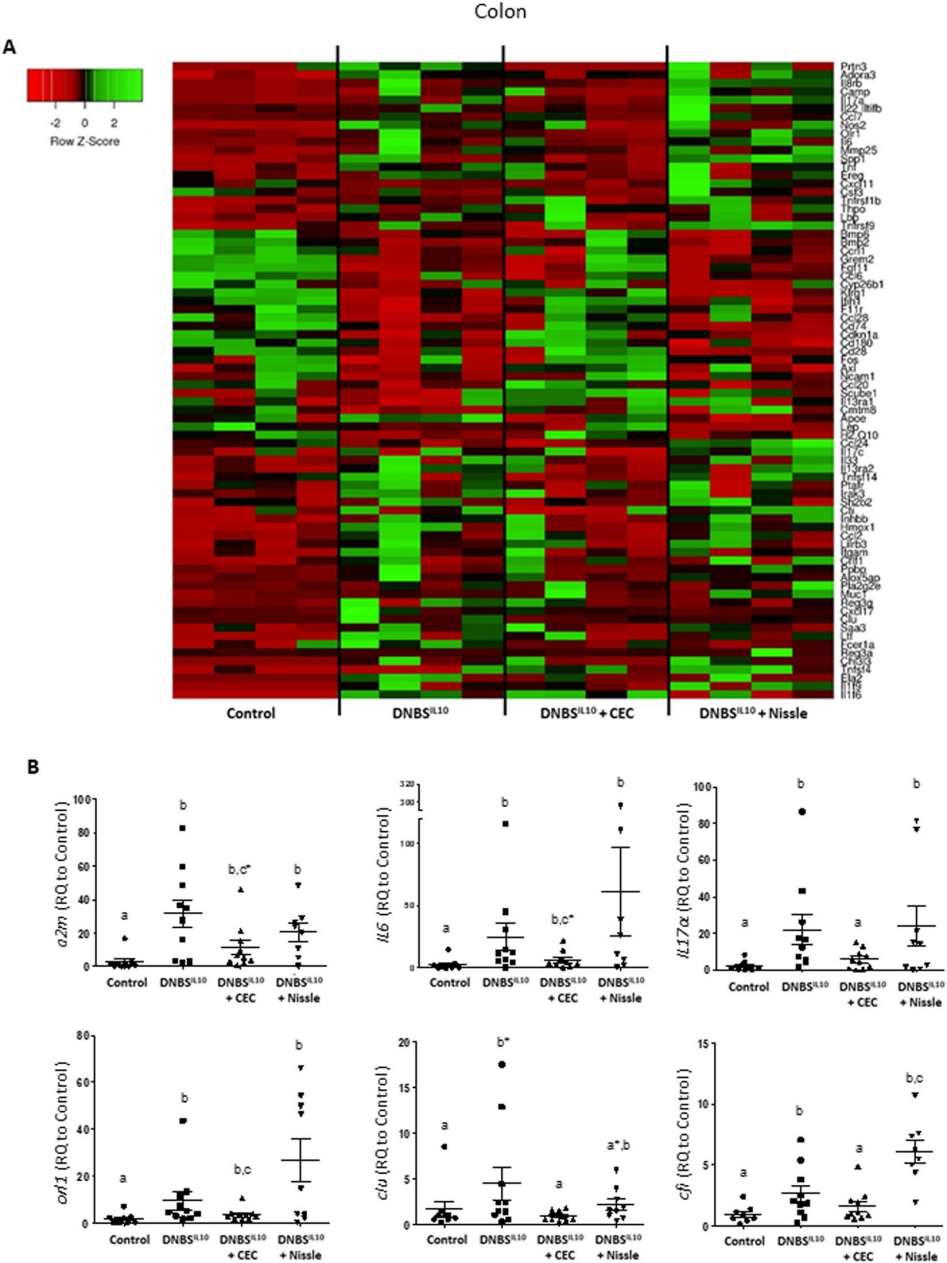
Supplementation of DNBS-treated IL10^−/−^ mice with CEC15 shifts colonic gene expression towards an anti-Inflammatory profile. Conventional IL10^−/−^ mice were treated intra-rectally with dinitrobenzene sulfonic acid (DNBS) and supplemented with CEC15 (DNBS^IL10^ + CEC), Nissle 1917 (DNBS^IL10^ + Nissle) or PBS (DNBS^IL10^), as a positive control for the disease; a group of mice, that received intra-rectally PBS instead of DNBS, was used as a negative control for the disease (Control). Gene expression profiling of the ileal mucosa was performed using the TaqMan Open Array system Mouse Inflammatory Panel. (**A**) Heatmap of the relative gene expression (Rq) to that of the Control group of the main modified genes of the colon. (**B**) Relative expression (Rq), measured with single TaqMan Assays, of *a2m* (*alpha-2-macroglobulin*), *IL6*, *IL17α*, *orl1* (oxidized low density lipoprotein receptor 1), *clu* (clusterin) and *cfi* (complement component factor I) in the colon, n = 8–10 mice/group. All values are presented as the means ± SEM. Mean values with letter designations are significantly different (non-parametric Mann-Whitney test).

We then analysed the inflammatory profile in Control (negative control for the disease), DNBS^IL10^, and DNBS^IL10^ + CEC/Nissle mice by focusing on a panel of genes involved in inflammation. Genes for which the resulting Rq values showed them to be significantly modified by DNBS treatment and rescued by *E*. *coli* strains, were selected and the corresponding Rq values plotted in a heatmap for the ileum and colon (Figs 6A and 7A).

The gene expression profile in the ileum of the control group was partially restored when DNBS^IL10^ mice were treated with the commensal *E*. *coli* strains CEC or Nissle (Fig. 6A). Thus, single TaqMan assay experiments confirmed that increased expression of interleukins such as *IL6*, *IL17α* and *ltf* (lactotransferrin) observed for DNBS^IL10^ relative to those of the control group was counteracted when mice received CEC or Nissle (Fig. 6B).

Similarly, the gene expression profile observed in the colon of DNBS^IL10^ + CEC was close to that of the control group (Fig. 7A). The effect of CEC was stronger than that of Nissle (Fig. 7A), with the recovery of Control expression levels for genes, such as the AMPs (*camp*, *pla2g2e*, *reg3α*, and *reg3β*) and several cytokines (*tnfα*, *cxcl11*, *IL22* and *IL6*), following *E*. *coli* treatment. Single TaqMan assays confirmed that the increased expression of the *a2m* (*alpha-2-macroglobulin*), *IL6*, *IL17α*, *orl1* (oxidized low density lipoprotein receptor 1), *clu* (clusterin) and *cfi* (complement component factor I) genes following DNBS treatment decreased to Control values when the DNBS treated mice received CEC (Fig. 7B).

### The effect of CEC on gene expression is independent of an effect on gut microbiota composition

**Figure 8:**
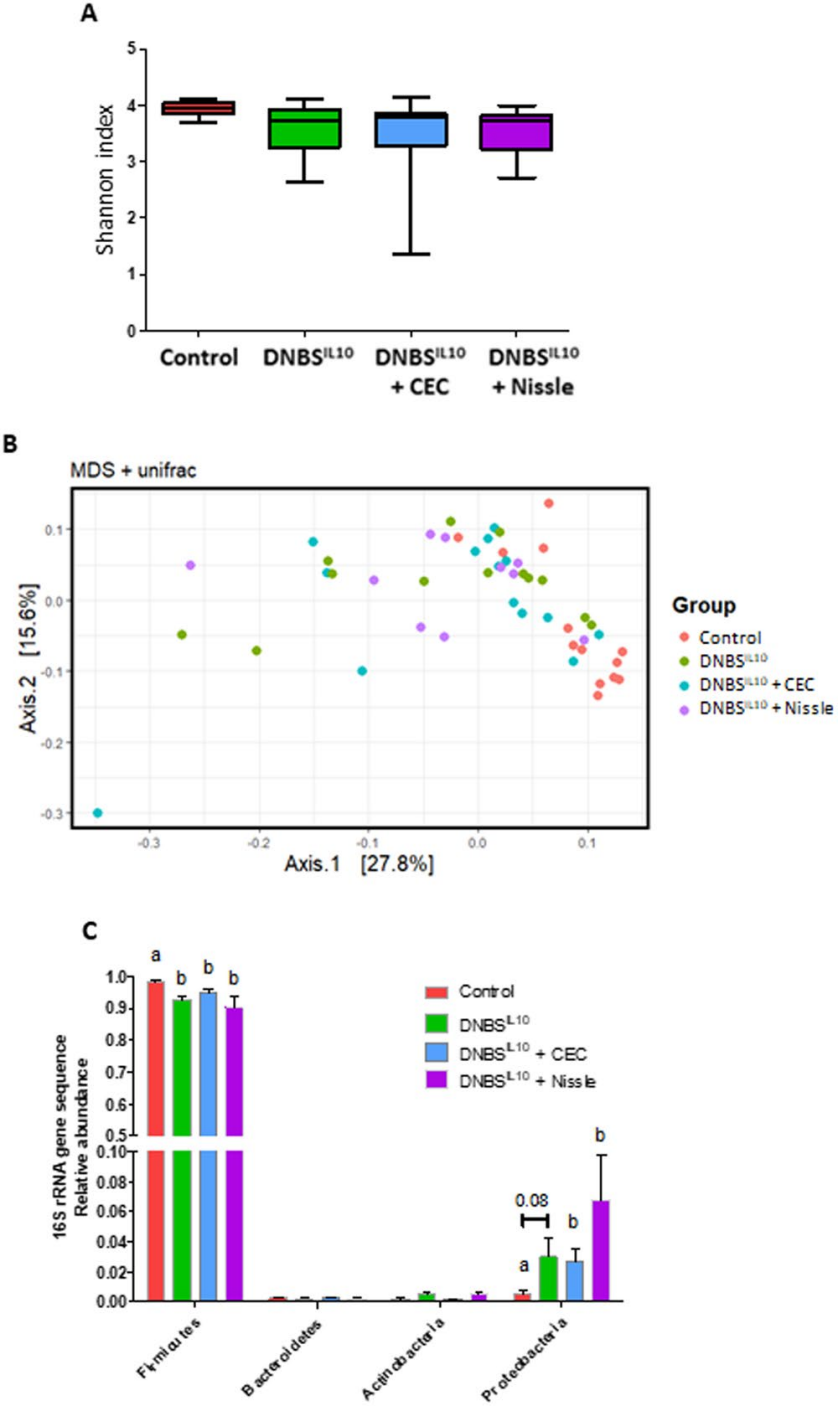
CEC15 supplementation does not affect the composition of the gut microbiota. Conventional IL10^−/−^ mice were treated intra-rectally with dinitrobenzene sulfonic acid (DNBS) and supplemented with CEC15 or Nissle 1917; PBS supplementation was used as a control. Analysis of the microbiota composition was performed by 16S rRNA gene sequencing of the cecal content. (**A**) α-diversity of the cecal microbiota measured with the Shannon index (a calculation method to measure the richness of the community (α-diversity) (**B**) β-diversity (measure of between-community diversity) of the cecal microbiota communities measured with the unweighted unifrac index. (**C**) Phylum abundance of Firmicutes, Bacteroidetes, Actinobacteria, and Proteobacteria. For all analyses n = 9–12 mice/group. All values are presented as the means ± SEM. Mean values with letter designations are significantly different (non-parametric Mann-Whitney test).

We investigated whether the effect of the *E*. *coli* strains involved modifications of the gut microbiota by analyzing the intestinal bacterial composition of the different experimental groups. We found no differences in the α-diversity, which measures the taxonomic richness of the cecal microbiota communities, between groups (Fig. 8A). In addition, β-diversity analysis, which measures the degree of similarity between the gut microbial communities, revealed no clustering of the mice according to the DNBS or *E*. *coli* treatments (Fig. 8B). At the phylum level, there was a decrease in the relative abundance of Firmicutes and an increase in that of Proteobacteria in the DNBS^IL10^ mice relative to that of the Control group, revealing dysbiosis in our model (Fig. 8C). Neither CEC nor Nissle treatment corrected the changes in microbiota composition induced by DNBS treatment.

## Discussion

Most studies demonstrating that several human diseases are associated with an expansion in the gut of *Enterobacteriaceae*, including *E*. *coli*, are based on sequencing data. However, this approach has insufficient resolution to detect genetically similar organisms of differing ecophysiology and impact on health. Considering phenotypic diversity at the strain level would aid the assessment of how resident *E*. *coli* may affect gut health and disease outcomes, as demonstrated by a recent study^30^.

Here, we extend our knowledge on a commensal *E*. *coli* strain that we previously isolated from suckling rodents, that we named CEC15, showing dynamic transcriptional responses of the ileum and the colon to CEC15. The expression of genes that play a key role in mucosal defence and the maintenance of mutualistic host-microbiota interactions was elevated in CEC mono-associated mice, similar to observations made with the probiotic Nissle 1917. Indeed, we show a core-response to both CEC and Nissle strains, in both the ileum and colon. This includes a set of genes involved in ROS turnover: *duox2* and *nox1*, both generating extracellular H_2_O_2_; the expression of *duoxa2*, required for the maturation of functional *duox2*, also increased, consistent with increased *duox2* expression. Expression of the AMPs, *reg3β* and *reg3ϒ*, *ang4*, and *pla2g2a* also increased, as well as that of *fut2*. We further demonstrated that components of the mucosal immune system recognize and react to the presence of commensal *E*. *coli* by increasing their expression, consistent with previous results that showed that GF mice colonized with members of the gut microbiota undergo immune system activation and development^31^.

Previous studies have shown that the intestinal expression of duox2^32^, members of the *reg3* family^33^, and *fut2*^34^ are upregulated by the gut microbiota in mice and that certain single commensal or probiotic bacterial strains can contribute to this effect^33–35^. These are key components of the innate immune system that help to fight against pathogens^36–38^ while preserving the symbiotic nature of the relationship between the gut microbiota and the host. In mice, *duox2*-generated H_2_O_2_ and members of the *reg3* family play a key role by spatially segregating indigenous bacteria, thereby dampening microbiota-induced mucosal immune responses^35,36^. *Fut2* is involved in the fucosylation of glycol-conjugates expressed on epithelial cells, providing a source of host-derived complex carbohydrates for the gut microbiota. The induction of *fut2* plays a role in colonization resistance against pathogens by restoring commensal diversity^38^.

Genes modulated in mono-associated mice include those that encode Ca^2+^-activated chloride channel 4, (*clca4*), the fructose transporter Slc2a5 (previously called GLUT5), and members of the water and glycerol channel aquaglyceroporin family (AQP -3 and -7), suggesting that the primary functions of the intestine may also be modulated in mono-associated mice^39^. *CLCA4* expression is downregulated in UC, suggesting that the control of electrolyte balance may be part of the defence mechanism against luminal microbes^40^. As recently shown, AQP3 transports H_2_O_2_ generated at the cell surface by NOX1 and DUOX2 to mediate signal transduction in colonic epithelia^41^. This further argues for a central role of *E*. *coli* strains on ROS turnover and signalling.

We tested the effect of the CEC strain in IL10^−/−^ mice, a well-recognized model for immune-mediated colitis, given its relevance to human IBD^42^. We speculate that the impact of the genetic background was low in the models we used. Indeed, the genetic background of wild type GF mice was C57BL/6 and that of GF IL10^−/−^ mice was a mix of C57BL/6 and 129/Ola. In our study, we found that, in the absence of IL10, the host response to commensal CEC and Nissle was stronger 21 days post-inoculation than in WT mice, as observed for *duox2*, *duox2a*, *nox1*, *nos2*, *reg3β*, *reg3ϒ*, and *fut2* expression. We also observed strong activation of immune responses, as observed for *cxcl10*, *tap1*, *il1β*, and *IL22*. In particular, neither the expression of *stat1* nor that of nod-like receptor 5 (*nlrc5*), were modified in WT mice by the presence of the two bacterial strains, whereas they were four to eight fold higher in mono-associated IL10^−/−^ than GF^IL10^ mice. A previous study suggested that IL10 may be involved in the control of the homeostatic relationship between indigenous strains of *E*. *coli* and the host^43^. In our study, intensification of the host response may have compensated for the absence of IL10.

We found that nos2 gene expression can be altered by the bacterial status of mice as seen in the difference between the CV^WT^ and GF^WT^ models. Both CEC and Nissle also upregulated nos2 expression in the WT mice but to a lesser extent than in the CV^WT^ group. This *E*. *coli* related increase of *nos2* is intensified in Il10^−/−^ mono-colonized mice. The role of intestinal *nos2* in *E*. *coli* growth has been demonstrated in an inflammation setting. Previous studies have shown an elevated intestinal expression of *nos2* during the inflammation process and that the host-nos2-derived by-products of reactive nitrogen species contribute to the proliferation of *E*. *coli*^44^. However, the physiological role of nos2 in this context has not been fully investigated and needs further studies.

We observed no evidences for intestinal inflammation in the mono-associated IL10^−/−^ mice, neither in the ileum or colon, despite a high level of colonization. Furthermore, the onset of inflammation was previously shown to be preceded by increased ileal and colonic permeability in the IL10^−/−^ mouse model^45^. In contrast, we found that some parameters of ileal and colonic permeability and integrity were improved in the CEC and Nissle mono-associated IL10^−/−^ groups, although we did not find any major differences in the passage of molecules. Thus, we found decreased electrical conductance associated with stronger staining of cadherin-1 in the ileum of IL10^−/−^ mice mono-colonised with *E*. *coli*. In addition, we found the epithelial integrity to be preserved, based on the positive effect of the strains on an epithelial proliferative marker. In addition, the ileal expression of Muc13 increased. Finally, we showed that CEC and Nissle can induce an increase in the thickness of the mucus layer in the ileum, which extended to the colon in the IL10^−/−^ mice.

We further investigated the effect of strain CEC in a disease setting using a model of conventional Il10^−/−^ mice exposed to DNBS. As previously mentioned, the IL-10 deficient mice model shares similarities with human IBD patients^42^. As the onset of colitis can vary among mice according to several factors^46^, a DNBS administration protocol, previously validated in our lab^25^, was used to synchronize colitis in this model. Our data show a beneficial role of CEC in promoting gut homeostasis upon mucosal injury in IL10^−/−^ mice. Although CEC was unable to reverse DNBS-associated weight loss, it attenuated several types of DNBS-induced damage, and reversed the gene expression profile towards that of the control group, both in the ileum and colon. The beneficial effect of CEC appeared to be stronger than that of the Nissle strain in the colon, based on the gene expression profile.

Several studies have reported that Nissle has an inhibitory effect on other *E. coli* species^47–49^. Regarding our 16S data, we did not observe any impact of CEC or Nissle on the gut microbiota composition. However, as 16S data analysis has insufficient resolution to distinguish genetically close organisms, we cannot exclude a remodelling of *E. coli* population by the administration of CEC15 or Nissle.

Our results that show a beneficial effect of CEC compared to those of other studies, strongly suggest that different *E*. *coli* strains of the gut microbiota may differ dramatically in their colitogenic or probiotic potential. For example, indigenous *E*. *coli* strains have a colitis-inducing potential in a genetically predisposed model for inflammation, whereas others have no negative impact^19^. The genetic determinants underlying such divergent behaviour are currently far from being understood. Preliminary data show that the genome of the CEC strain does not contain the genomic *pks* island (data not shown). This is in contrast to the Nissle strain, for which the *pks* island was shown. This gene cluster encodes non-ribosomal peptide synthetases (NRPS) and polyketide synthetases (PKS) and produces colibactin (a peptide-polyketide genotoxin) that can induce DNA damage by inducing double-strand breaks^50^. However, the role of *pks* in gut health is a matter of debate. Indeed, the probiotic properties of Nissle are linked to the *pks* island, more precisely to a lipopeptide encoded in this this region^51^. Sequencing of the CEC15 genome and genomic comparison of other commensal *E*. *coli* strains with a known phenotype will help to identify the molecular signatures that can distinguish *E. coli* strain impact. Preliminary analysis, based on a PCR method enabling an *E*. *coli* isolate to be assigned to one of the *E*. *coli* phylogroups^52^ reveals that the CEC15 belongs to the phylogroup C, a group of strains closely related to the phylogroup B1. So, interestingly, CEC15, which had a stronger anti-inflammatory effect than Nissle, belongs to a different phylogroup (Phylogroup B2 for Nissle^53^).

Recently, a study identified a respiratory pathway that is only operational during episodes of inflammation and that can drive *Enterobacteriaceae* expansion during colitis^54^. Selective inhibition of this pathway prevents dysbiotic overgrowth of *Enterobacteriaceae*, including *E*. *coli*, in murine models of colitis^55^. The implementation of this strategy, based on a better knowledge of the effect of commensal *E*. *coli*, has the potential to control the population of *E*. *coli*, making it possible to discard potential harmful microbes while preserving the potentially beneficial ones.

## Supplementary information

supplementary data

Supplementary Table 1-2

## Data Availability

Full data will be available from the corresponding author on reasonable request.
