# Supplementary material for: The commensal Escherichia coli CEC15 reinforces intestinal defences in gnotobiotic mice and is protective in a chronic colitis mouse model
Source: Sci Rep. 2019 Aug 7;9:11431. doi: 10.1038/s41598-019-47611-9 (PMC6685975; doi:10.1038/s41598-019-47611-9)
Supplement: Supplementary file 1 — supplementary data [file 41598_2019_47611_MOESM1_ESM.pdf]

Supplementary Fig 1 - 10

The commensal *Escherichia coli* CEC15 reinforces intestinal defences in gnotobiotic mice and is protective in a chronic colitis mouse model

Unai Escribano-Vazquez, Sophie Verstraeten, Rebeca Martin, Florian Chain, Philippe Langella, Muriel Thomas , and Claire Cherbuy

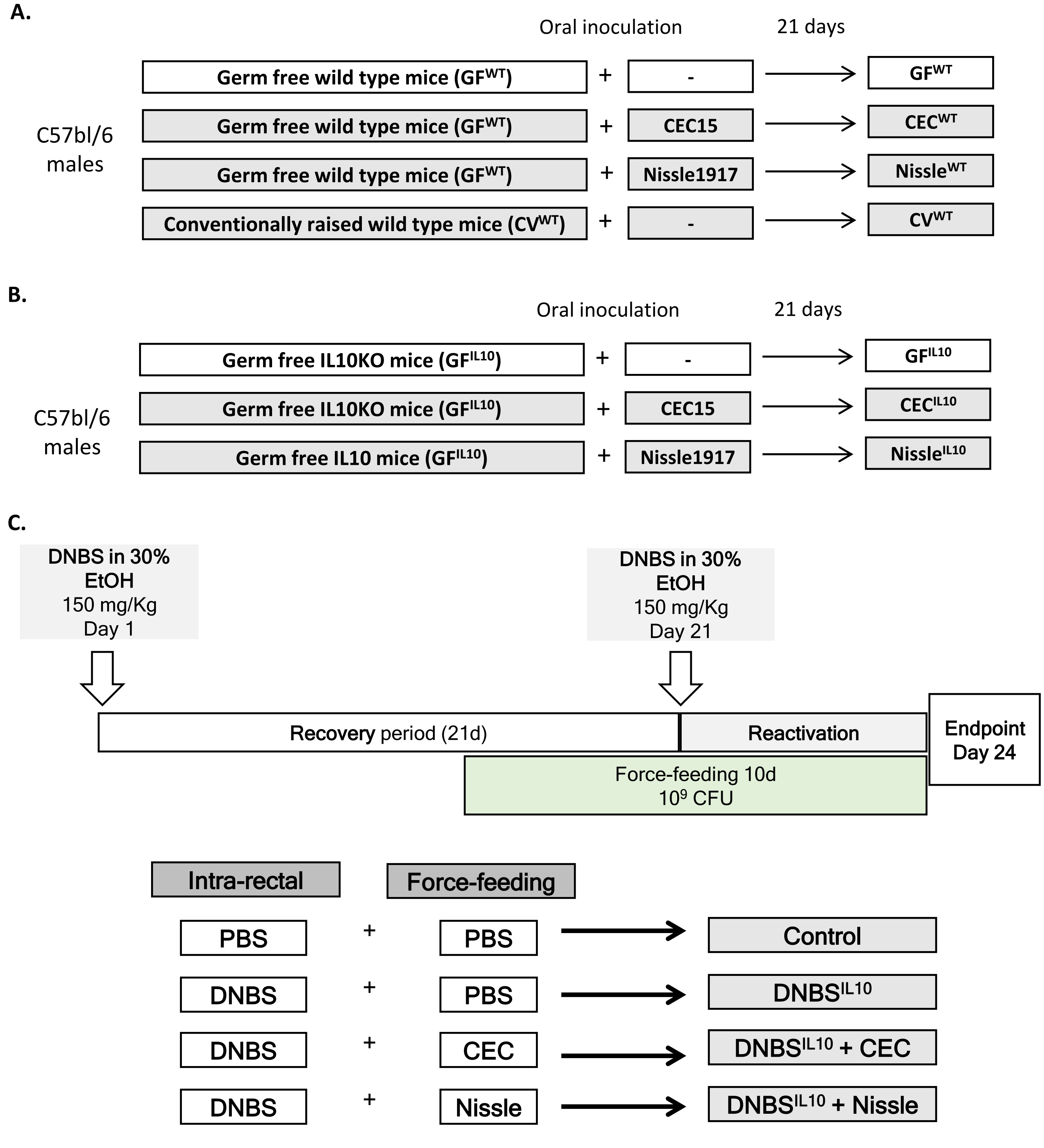

Supplementary Figure 1. Experimental design. (**A, B**) Male C57bl/6 wild-type (WT) or $IL10^{-/-}$ mice were kept germ free (GF^{WT}, GF^{IL10}) or mono-colonized with the *E. coli* CEC15 (CEC^{WT}; CEC^{IL10}) or Nissle 1917 ($Nissle^{WT}$; $Nissle^{IL10}$) strain for 21 days. All mice were 8 to 10 weeks old at the time of inoculation and were sacrificed 21 days post-inoculation, i.e. at 10-12 weeks of age (**C**) Conventional $IL10^{-/-}$ mice treated intra-rectally with dinitrobenzene sulfonic acid (DNBS) and supplemented with CEC15 or Nissle 1917: Control, $DNBS^{IL10}$, $DNBS^{IL10}$ + CEC, and $DNBS^{IL10}$ + Nissle. Experimental chronic colitis was induced in six-to-eight-week-old mice. A dose of 1 x 10^9 CFU of CEC ($DNBS^{IL10}$ + CEC) or Nissle ($DNBS^{IL10}$ + Nissle) were given daily by oral gavage ten days before sacrifice. Positive control group for the disease ($DNBS^{IL10}$) received the same volume of PBS.

A. Ileum

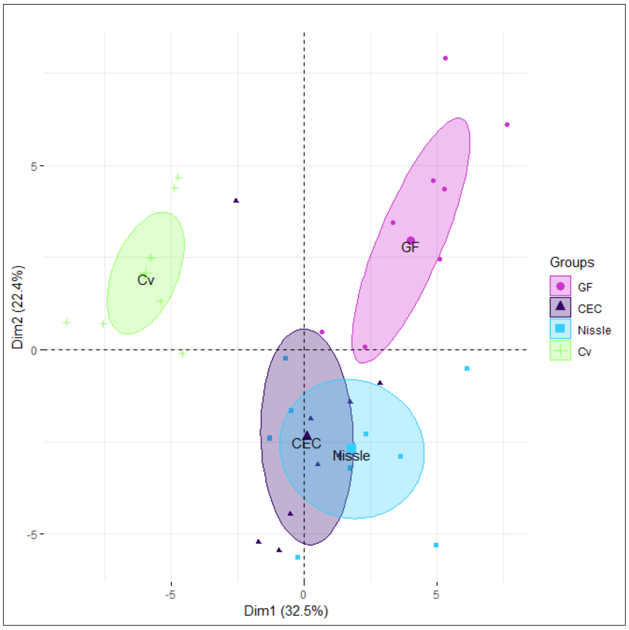

B. Colon

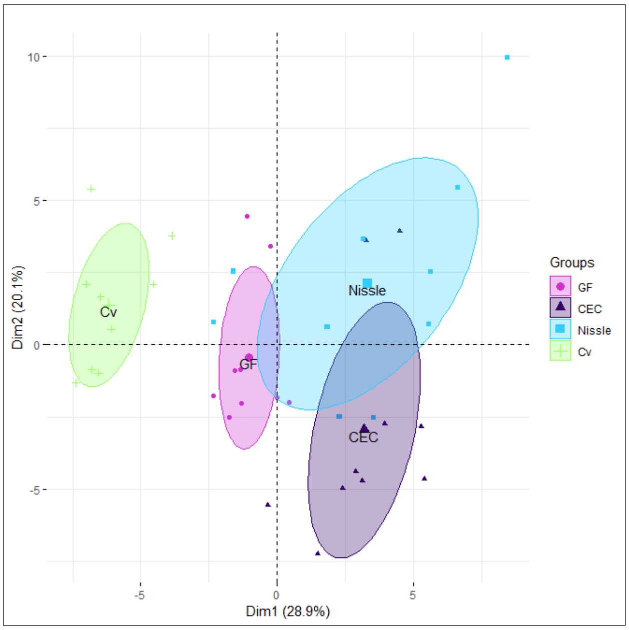

Supplementary Figure 2. Principal component analyses of ileal and colonic gene expression of CEC15 or Nissle 1917 mono-associated WT mice relative to that of GF^{WT} and CV^{WT} mice. Gene expression profiling of the ileum (A) and colon (B) of germ-free wild-type mice (GF^{WT}) colonized for 21 days with the CEC15 (CEC^{WT}) or Nissle 1917 ($Nissle^{WT}$) strain and conventional (CV^{WT}) mice was carried out with the TaqMan OpenArray system. Principal component analyses (PCA) was performed on the relative gene expression (Rq) of the different groups to that of the GF^{WT} group; n = 9 - 10 mice/group.

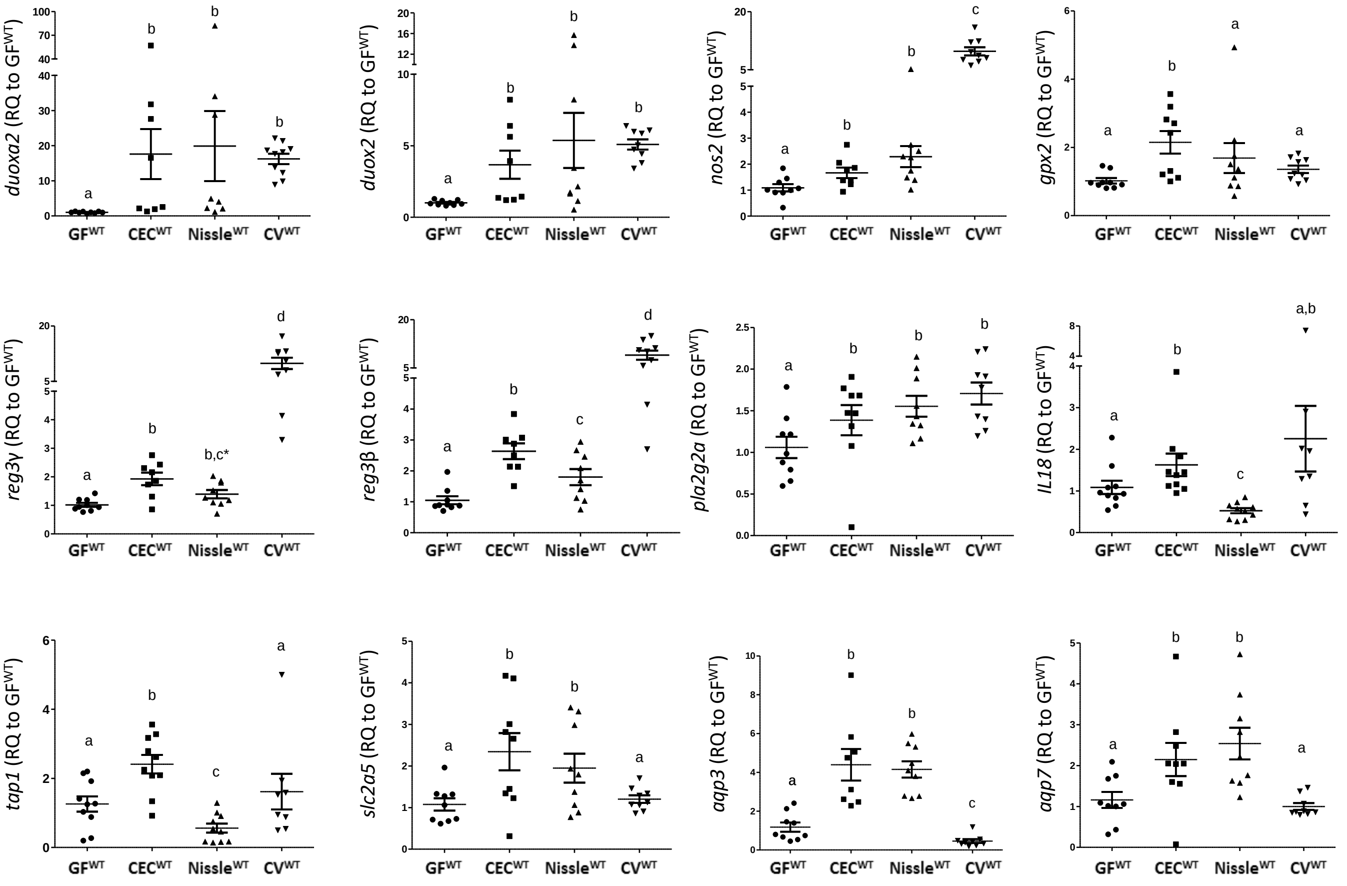

Supplementary Figure 3. Selection of ileal genes for which the expression is modified by CEC15 or Nissle 1917 in WT gnotobiotic mice. Gene expression analyses of the ileal mucosa with the single TaqMan Assays of germ-free wild-type mice (GF^{WT}) colonized for 21 days with the CEC15 (CEC^{WT}) or Nissle 1917 ($Nissle^{WT}$) strain. Relative gene expression (Rq) of the various groups to that of the germfree (GF^{WT}) group for the ileum; n = 7-10 mice/group. All values are presented as the means ± SEM. Mean values with letter designations are significantly different.

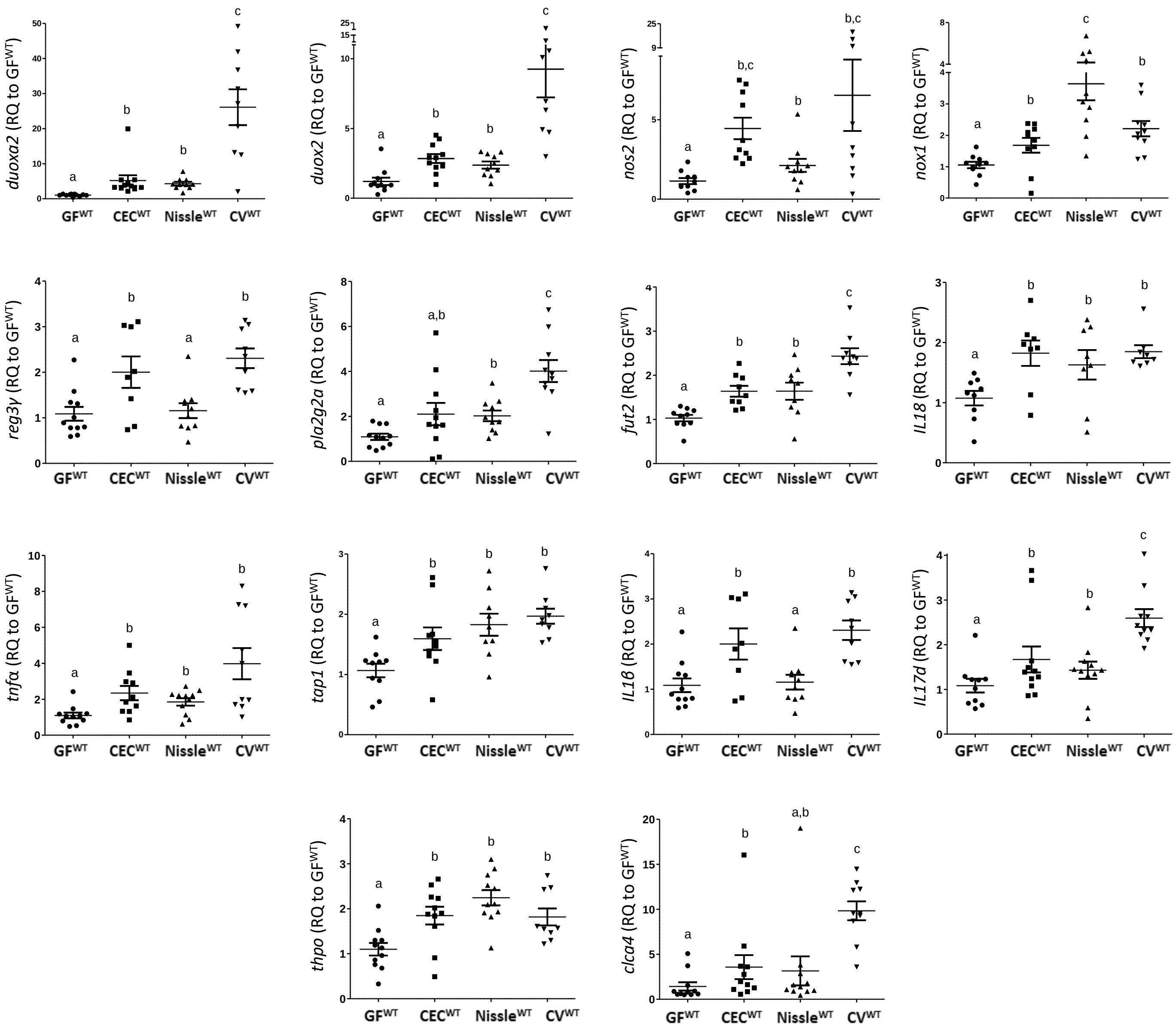

Supplementary Figure 4. Selection of colonic genes for which the expression is modified by CEC15 or Nissle 1917 in WT gnotobiotic mice. Gene expression analyses of the colonic mucosa with the single TaqMan Assays of germ-free wild-type mice (GF^{WT}) colonized for 21 days with the CEC15 (CEC^{WT}) or Nissle 1917 ($Nissle^{WT}$) strain. Relative gene expression (Rq) of the various groups to that of the germfree (GF^{WT}) group for the colon; n = 7-10 mice/group. All values are presented as the means ± SEM. Mean values with letter designations are significantly different.

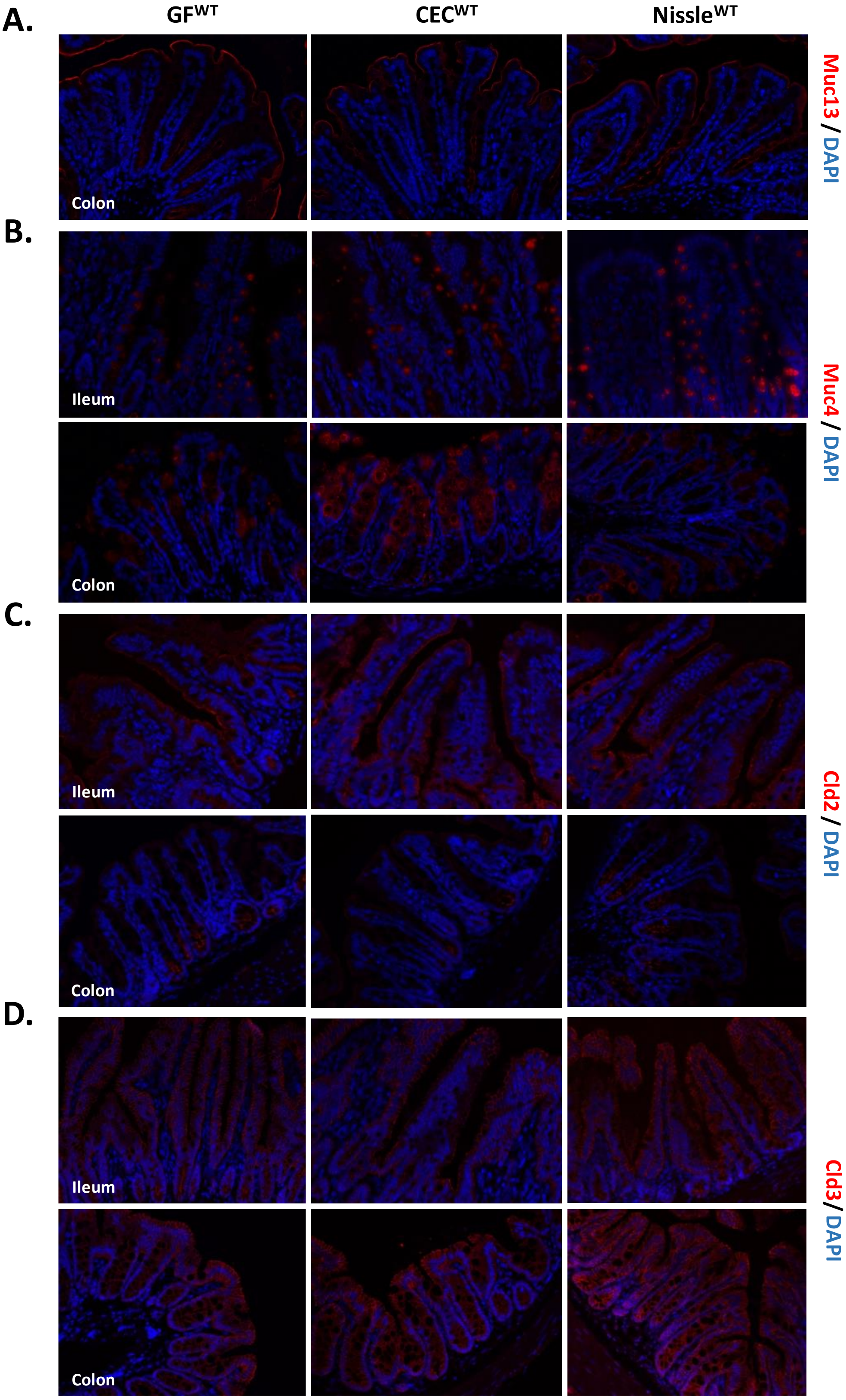

Supplementary Figure 5. Analysis of intestinal barrier proteins of CEC15 or Nissle 1917 mono-colonized WT mice. Germ-free wild-type mice (GF^{WT}) were colonized for 21 days with the CEC15 (CEC^{WT}) or Nissle 1917 ($Nissle^{WT}$) strain. Immunostaining of the intestinal epithelium proteins mucin 13 (**A**, red), mucin 4 (**B**, red; Santa Cruz; 1/500), claudin-2 (**C**, red; Invitrogen; 1/1000), claudin-3 (**D**, red; Abcam; 1/100), and the cell nuclei (blue, DAPI) of the ileum and/or colon.

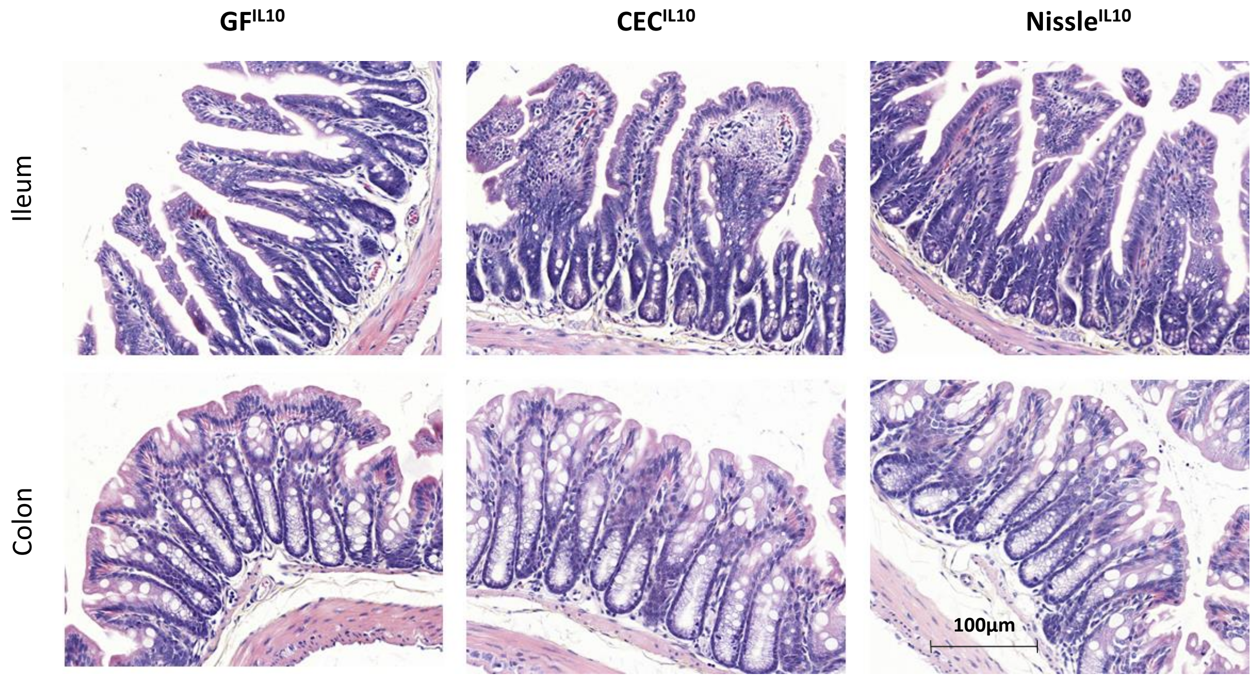

Supplementary Figure 6. Histological analyses of mono-associated IL10$^{-/-}$ mice. Germ-free IL10$^{-/-}$ mice (GF^{IL10}) were colonized for 21 days with the CEC15 (CEC^{IL10}) or Nissle 1917 (Nissle^{IL10}) strain. Hematoxylin eosin staining of the ileum and colon was carried out.

A.

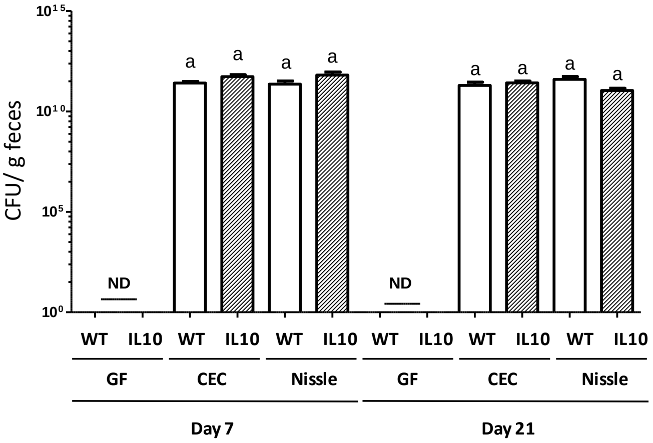

Supplementary Figure 7. Colonization of mono-associated WT and IL10$^{-/-}$ mice by CEC15 or Nissle 1917. Fecal and caecal samples were collected 7 and 21 days post-inoculation with *E. coli*, respectively. DNA was extracted as described in [1] for *E. coli* enumeration. The qPCR was performed using the method described in [2] with *E. coli*-specific primers (Fwd_E.coli CATGCCGCGTGTATGAAGAA; Rv_E.coli CGGGTAACGTCAATGAGCAAA). Standard curves generated from 10-fold serial dilutions of DNA samples of specific strains were used for quantification. Bacterial equivalents were interpolated from a standard curve generated in the same experiment using cycle threshold values in the linear range. Results are expressed as colony-forming unit (CFU) equivalents per gram of content.

1. Tomas, J., et al., *Primocolonization is associated with colonic epithelial maturation during conventionalization.* FASEB J, 2013. 27(2): p. 645-55.
2. Mayeur, C., et al., *Faecal D/L lactate ratio is a metabolic signature of microbiota imbalance in patients with short bowel syndrome.* PLoS One, 2013. 8(1): p. e54335.

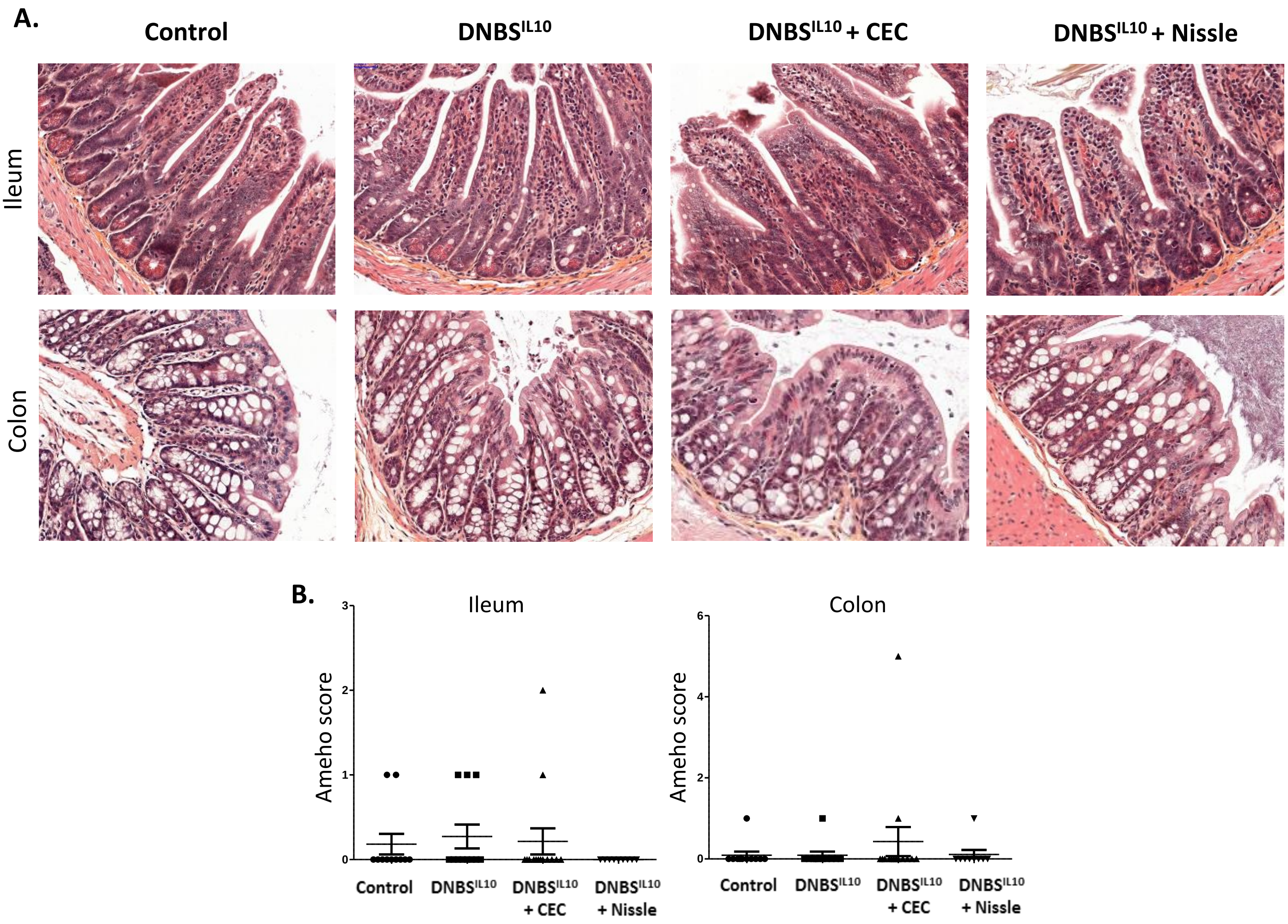

Supplementary Figure 8. Assessment of the Ameho score in the chronic colitis model, conventional IL10$^{-/-}$ DNBS-treated mice. Conventional IL10$^{-/-}$ mice were treated intra-rectally with dinitrobenzene sulfonic acid (DNBS) or PBS (Control) and supplemented with CEC15 (DNBS^{IL10}+ CEC), Nissle 1917 (DNBS^{IL10}+ Nissle) or PBS (DNBS^{IL10}). Ameho score is determined according to the following classification: 0 = non-histological modifications, when compared to healthy control; 1 = inflammatory infiltration into the mucosa, capillary proliferation / muscular-mucosal intact; 2 = grade 1 but which affects > 50% of the cut; 3 = inflammatory infiltration + edema + ulcer in the muscular-mucosa / no necrosis; 4 = grade 3 but which affects > 50% of the cut; 5 = necrosis; 6 = grade 5 but which affects > 50% of the cut; n = 11 - 14 mice/group were analyzed for the Ameho score. All values are presented as the means ± SEM. Mean values with letter designations are significantly different.

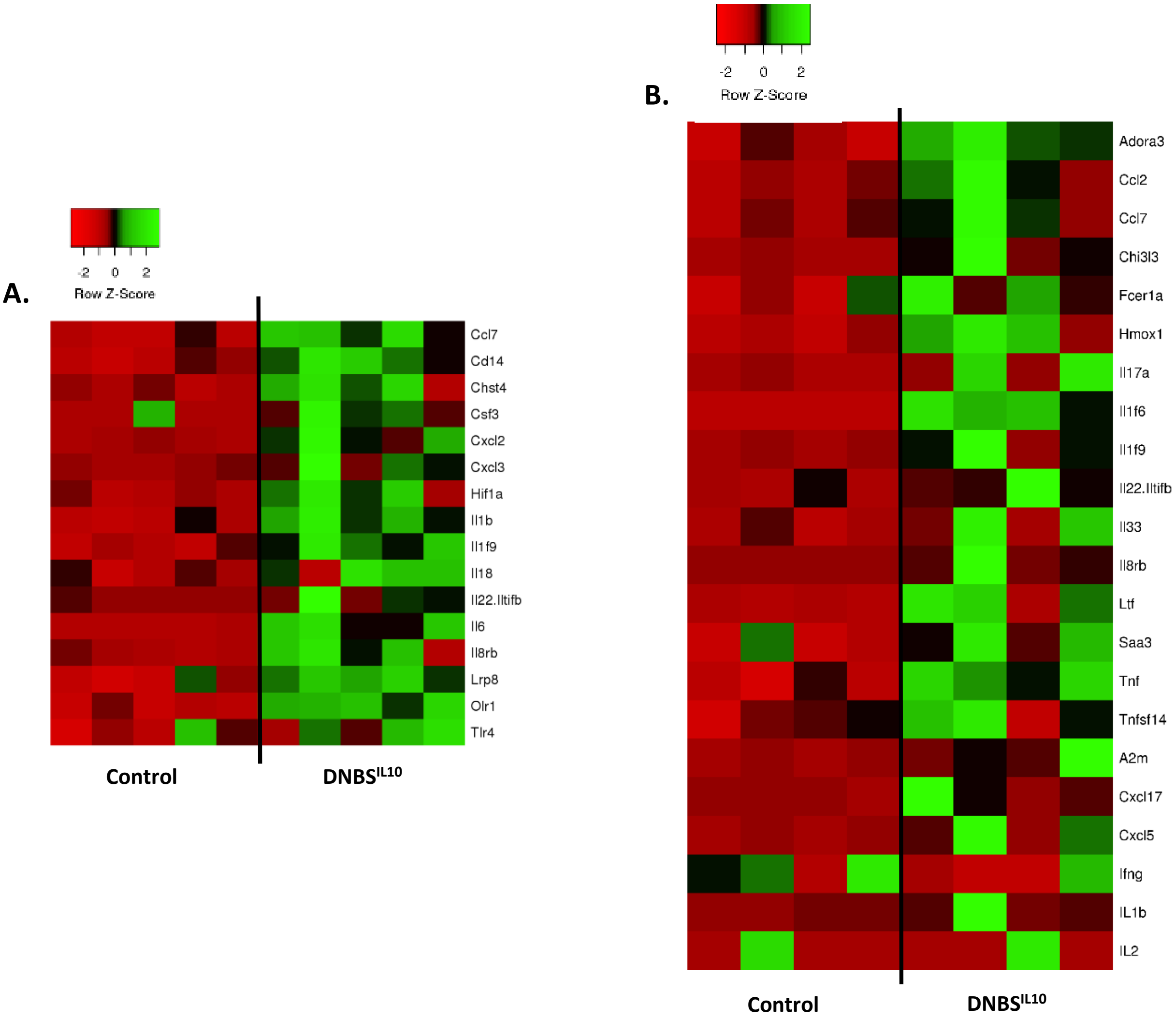

Supplementary Figure 9. Intestinal gene expression profiles induced during DNBS treatment of conventional IL10$^{-/-}$ mice. Conventional IL10$^{-/-}$ mice were treated intra-rectally with dinitrobenzene sulfonic acid (DNBS^{IL10}) or PBS, used as negative control (Control). Gene expression profiling of the ileal and colonic mucosa was performed with the Taqman Open Array system Mouse Inflammatory Panel. (**A**) and (**B**): Heatmaps of the relative gene expression (Rq) to that of the Control group of the main modified genes of the ileum and colon, respectively.

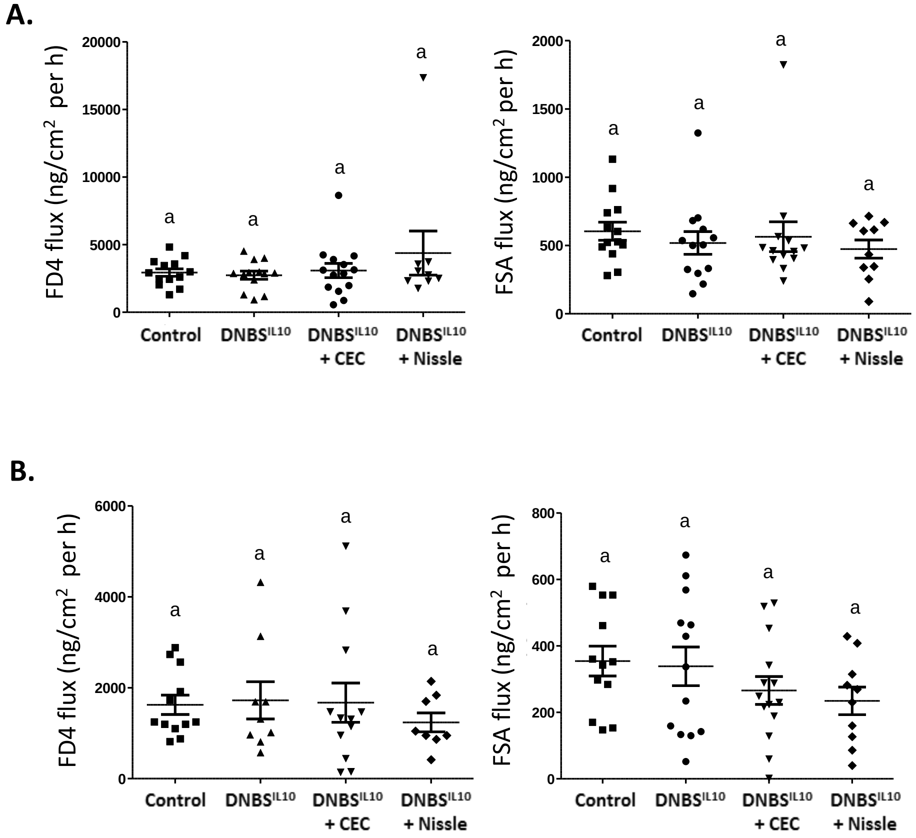

Supplementary Figure 10. *Ex vivo* intestinal permeability in the chronic colitis model, conventional IL10$^{-/-}$ DNBS-treated mice. Conventional IL10$^{-/-}$ mice were treated intra-rectally with dinitrobenzene sulfonic acid (DNBS) or PBS (Control) and supplemented with CEC15 (DNBS^{IL10}+ CEC), Nissle 1917 (DNBS^{IL10}+ Nissle) or PBS (DNBS^{IL10}). (**A**) and (**B**): analysis of *ex vivo* para-cellular permeability using the Ussing chamber system with FITC-dextran (4 KDa; FD4) and FITC-sulfonic acid (400 Da; FSA) for the ileum (**A**) and colon (**B**), n= 5-17 mice per group.
