# Supplementary material for: The commensal Escherichia coli CEC15 reinforces intestinal defences in gnotobiotic mice and is protective in a chronic colitis mouse model
Source: Sci Rep. 2019 Aug 7;9:11431. doi: 10.1038/s41598-019-47611-9 (PMC6685975; doi:10.1038/s41598-019-47611-9)
Supplement: Supplementary file 2 — Supplementary Table 1-2 [file 41598_2019_47611_MOESM2_ESM.zip]

Supplementary Table 1-2

**The commensal *Escherichia* *coli* CEC15 reinforces intestinal defences in gnotobiotic mice and is protective in a chronic colitis mouse model**

Unai Escribano-Vazquez, Sophie Verstraeten, Rebeca Martin, Florian Chain, Philippe Langella, Muriel Thomas , and Claire Cherbuy

Table 1. TaqMan® OpenArray® Real-Time PCR Custom Assays Format of the ileum

| **Gene** | **Assay ID** |
| --- | --- |
| Abca7 | Mm00497010_m1 |
| Abcd1 | Mm00431749_m1 |
| Abcg8 | Mm00445980_m1 |
| Actb | Mm00607939_s1 |
| Adh1 | Mm00507711_m1 |
| Aldh18a1 | Mm00444767_m1 |
| Aldh3a2 | Mm00839320_m1 |
| Aldh9a1 | Mm00480240_m1 |
| Alpi | Mm01285814_g1 |
| Ang4 | Mm01315577_s1 |
| Angptl4 | Mm00480431_m1 |
| Ano10 | Mm00661819_m1 |
| Ano6 | Mm00614693_m1 |
| Apaf1 | Mm01223701_m1 |
| Aqp1 | Mm01326466_m1 |
| Aqp11 | Mm00613023_m1 |
| Aqp3 | Mm01208559_m1 |
| Aqp7 | Mm00431839_m1 |
| Areg | Mm01354339_m1 |
| Ascl2 | Mm01268891_g1 |
| Axin2 | Mm00443610_m1 |
| B3gnt2 | Mm00479497_s1 |
| B3gnt7 | Mm00507533_m1 |
| B4galt1 | Mm00480752_m1 |
| B4galt5 | Mm00480147_m1 |
| B4galt6 | Mm00480045_m1 |
| Bmp1 | Mm00802220_m1 |
| Cat | Mm00437992_m1 |
| Ccl19 | Mm00839967_g1 |
| Ccl25 | Mm00436443_m1 |
| Ccl3 | Mm00441259_g1 |
| Ccl6 | Mm01302419_m1 |
| Ccna2 | Mm00438063_m1 |
| Ccnd1 | Mm00432359_m1 |
| Ccne1 | Mm01266311_m1 |
| Cd55 | Mm00438377_m1 |
| Cdc25a | Mm00483162_m1 |
| Cdc25b | Mm00499136_m1 |
| Cdh1 | Mm01247357_m1 |
| Cdk4 | Mm00726334_s1 |
| Ceacam1 | Mm04204476_m1 |
| Cebpb | Mm00843434_s1 |
| Cftr | Mm00445197_m1 |
| Clca4a | Mm01614382_m1 |
| Cldn15 | Mm00517635_m1 |
| Cldn18 | Mm00517321_m1 |
| Cldn2 | Mm00516703_s1 |
| Cldn3 | Mm00515499_s1 |
| Cldn4 | Mm00515514_s1 |
| Cldn7 | Mm00516817_m1 |
| Cldn8 | Mm00516972_s1 |
| Cox18 | Mm01344766_m1 |
| Cox7a2 | Mm00438299_m1 |
| Cox8a | Mm02342396_g1 |
| Cxcl10 | Mm00445235_m1 |
| Cyp2c55 | Mm00472168_m1 |
| Cyp2d26 | Mm00472520_m1 |
| Cyp3a13 | Mm00484110_m1 |
| Cyp4b1 | Mm00484138_m1 |
| Cyp4f14 | Mm00491623_m1 |
| Cyp4v3 | Mm00530567_m1 |
| Ddx5 | Mm00833270_gH |
| Duox2 | Mm01326247_m1 |
| Duoxa2 | Mm00470560_m1 |
| E2f1 | Mm00432939_m1 |
| Egfr | Mm00433023_m1 |
| Egr1 | Mm00656724_m1 |
| Eng | Mm00468252_m1 |
| Epcam | Mm00493214_m1 |
| F11r | Mm00554113_m1 |
| Fgfbp1 | Mm00456064_s1 |
| Fos | Mm00487425_m1 |
| Fut2 | Mm00490152_s1 |
| Gapdh | Mm99999915_g1 |
| Gata3 | Mm00484683_m1 |
| Ghr | Mm00439093_m1 |
| Gpr35 | Mm01973686_s1 |
| Gpx2 | Mm00850074_g1 |
| Gss | Mm00515065_m1 |
| Gsta4 | Mm00494803_m1 |
| Gstm2 | Mm00725711_s1 |
| Gstm6 | Mm00656783_gH |
| Gstp1 | Mm04213618_gH |
| Gstt1 | Mm00492506_m1 |
| Gusb | Mm00446953_m1 |
| Hif1a | Mm00468869_m1 |
| Hif3a | Mm00469375_m1 |
| Hnf1a | Mm00493434_m1 |
| Hpgd | Mm00515121_m1 |
| Ifng | Mm99999071_m1 |
| Igf2bp2 | Mm00805575_m1 |
| Igfbp3 | Mm01187817_m1 |
| Il10 | Mm01288386_m1 |
| Il10ra | Mm00434151_m1 |
| Il12a | Mm00434169_m1 |
| Il12b | Mm01288989_m1 |
| Il17d | Mm01313472_m1 |
| Il17ra | Mm00434214_m1 |
| Il17rc | Mm00506606_m1 |
| Il18 | Mm00434226_m1 |
| Il1b | Mm01336189_m1 |
| Il22 | Mm00444241_m1 |
| Il23a | Mm00518984_m1 |
| Il2rb | Mm00434268_m1 |
| Il2rg | Mm00442885_m1 |
| Il33 | Mm00505403_m1 |
| Il3ra | Mm00434273_m1 |
| Il4ra | Mm00439634_m1 |
| Il6 | Mm00446190_m1 |
| Il6ra | Mm00439653_m1 |
| Insig1 | Mm00463389_m1 |
| Insr | Mm01211875_m1 |
| Jun | Mm00495062_s1 |
| Klf4 | Mm00516104_m1 |
| Klf5 | Mm00456521_m1 |
| Krt20 | Mm00508106_m1 |
| Lbp | Mm00493139_m1 |
| Lcn2 | Mm01324470_m1 |
| Lgr5 | Mm00438890_m1 |
| Lrp1 | Mm00464608_m1 |
| Lrp6 | Mm00999795_m1 |
| Lyz1 | Mm00657323_m1 |
| Lyz2 | Mm01612741_m1 |
| Marveld2 | Mm01282909_m1 |
| Mif | Mm01611157_gH |
| Mki67 | Mm01278617_m1 |
| Mmp15 | Mm00485062_m1 |
| Mmp7 | Mm00487724_m1 |
| Mpo | Mm01298424_m1 |
| Muc1 | Mm00449604_m1 |
| Muc13 | Mm00495397_m1 |
| Muc2 | Mm01276696_m1 |
| Muc4 | Mm00466886_m1 |
| Myc | Mm00487804_m1 |
| Myd88 | Mm00440338_m1 |
| Mylk | Mm00653039_m1 |
| Nab1 | Mm01257272_m1 |
| Naip1 | Mm00657763_gH |
| Naip5 | Mm00783826_s1 |
| Nat8 | Mm00517808_m1 |
| Ndst1 | Mm00447005_m1 |
| Nfkbiz | Mm00600522_m1 |
| Nlrc5 | Mm01243039_m1 |
| Nlrp3 | Mm00840904_m1 |
| Nlrp6 | Mm00460229_m1 |
| Nlrp9b | Mm01312681_g1 |
| Nlrx1 | Mm00617978_m1 |
| Nod1 | Mm00805062_m1 |
| Nod2 | Mm00467543_m1 |
| Nos2 | Mm00440502_m1 |
| Nox1 | Mm00549170_m1 |
| Noxa1 | Mm00549172_m1 |
| Nqo1 | Mm01253561_m1 |
| Nqo2 | Mm01332867_m1 |
| Nrtn | Mm03024002_m1 |
| Oas1a | Mm00836412_m1 |
| Ocln | Mm00500912_m1 |
| Ogt | Mm00507317_m1 |
| Pcna | Mm00448100_g1 |
| Pglyrp1 | Mm00437150_m1 |
| Pigr | Mm00465049_m1 |
| Pla2g16 | Mm01231746_m1 |
| Pla2g2a | Mm00448160_m1 |
| Pla2g6 | Mm01299491_m1 |
| Plaa | Mm01256059_m1 |
| Pofut1 | Mm00475567_m1 |
| Pparg | Mm00440940_m1 |
| Prdx1 | Mm01621996_s1 |
| Rdh7 | Mm00489956_g1 |
| Reg1 | Mm00485651_m1 |
| Reg3b | Mm00440616_g1 |
| Reg3g | Mm00441127_m1 |
| Reg4 | Mm00471115_m1 |
| Retnlb | Mm00445845_m1 |
| Ripk2 | Mm00446816_m1 |
| Ripk3 | Mm00444947_m1 |
| Rorc | Mm01261022_m1 |
| S100a6 | Mm00771682_g1 |
| S100a8 | Mm01220132_g1 |
| S100a9 | Mm00656925_m1 |
| Slc11a2 | Mm00435363_m1 |
| Slc12a2 | Mm01265951_m1 |
| Slc15a3 | Mm00491666_m1 |
| Slc23a1 | Mm00495520_m1 |
| Slc25a11 | Mm00452509_m1 |
| Slc25a44 | Mm00618558_m1 |
| Slc25a5 | Mm00846873_g1 |
| Slc27a2 | Mm00449517_m1 |
| Slc2a5 | Mm00600311_m1 |
| Slc5a11 | Mm00461434_m1 |
| Slc5a4b | Mm00452283_m1 |
| Slc7a1 | Mm01219063_m1 |
| Slc7a7 | Mm00448764_m1 |
| Slc7a8 | Mm01318974_m1 |
| Slc9a1 | Mm00444270_m1 |
| Socs1 | Mm00782550_s1 |
| Sod2 | Mm01313000_m1 |
| Sod3 | Mm01213380_s1 |
| Sord | Mm00455377_g1 |
| Sox2 | Mm03053810_s1 |
| Sprr1a | Mm01962902_s1 |
| Sprr2a1 | Mm00845122_s1 |
| St3gal1 | Mm00501493_m1 |
| Stat1 | Mm01257286_m1 |
| Stat6 | Mm01160477_m1 |
| Sulf2 | Mm01248029_m1 |
| Tap1 | Mm00443188_m1 |
| Tbp | Mm00446973_m1 |
| Tff2 | Mm00447491_m1 |
| Tff3 | Mm00495590_m1 |
| Tgfbi | Mm01337605_m1 |
| Thpo | Mm00437040_m1 |
| Tjp1 | Mm00493699_m1 |
| Tjp2 | Mm00495620_m1 |
| Tlr4 | Mm00445273_m1 |
| Tlr5 | Mm00546288_s1 |
| Tlr9 | Mm00446193_m1 |
| Tnf | Mm00443258_m1 |
| Trp53 | Mm01731290_g1 |
| Txnip | Mm01265659_g1 |
| Ubc | Mm01201237_m1 |
| Ugt1a1 | Mm02603337_m1 |
| Ugt2b36 | Mm01615926_m1 |
| Ugt2b5 | Mm01623253_s1 |

Table 2. TaqMan® OpenArray® Real-Time PCR Custom Assays Format of the colon

| **Gene** | **Assay ID** |
| --- | --- |
| Abca1 | Mm00442646_m1 |
| Abcb1a | Mm00440761_m1 |
| Abcb1b | Mm01324115_m1 |
| Abcc1 | Mm00456156_m1 |
| Abcc3 | Mm00551550_m1 |
| Actb | Mm00607939_s1 |
| Adipoq | Mm00456425_m1 |
| Aim2 | Mm01295719_m1 |
| Alox5 | Mm01182749_m1 |
| Ang4 | Mm01315577_s1 |
| Ano1 | Mm00724407_m1 |
| Ano5 | Mm00624629_m1 |
| Ano6 | Mm00614693_m1 |
| Areg | Mm01354339_m1 |
| Arntl | Mm00500226_m1 |
| Axin2 | Mm00443610_m1 |
| B4galnt2 | Mm00484661_m1 |
| B4galt1 | Mm00480752_m1 |
| Best2 | Mm00520372_m1 |
| Bmp5 | Mm00432091_m1 |
| Bmpr1b | Mm00432117_m1 |
| Brinp3 | Mm00463491_m1 |
| C1galt1 | Mm01167001_m1 |
| Ccbl1 | Mm00549584_m1 |
| Ccbl2 | Mm00620553_m1 |
| Ccl19 | Mm00839967_g1 |
| Ccl6 | Mm01302419_m1 |
| Ccl8 | Mm01297183_m1 |
| Ccl9 | Mm00441260_m1 |
| Ccna2 | Mm00438063_m1 |
| Ccnd2 | Mm00438070_m1 |
| Ccnd3 | Mm01612362_m1 |
| Ccndbp1 | Mm00487558_m1 |
| Ccne1 | Mm01266311_m1 |
| Cd28 | Mm00483137_m1 |
| Cd40 | Mm00441891_m1 |
| Cdc25a | Mm00483162_m1 |
| Cdc25b | Mm00499136_m1 |
| Cdk1 | Mm00772472_m1 |
| Cebpa | Mm00514283_s1 |
| Cftr | Mm00445197_m1 |
| Chkb | Mm04213225_s1 |
| Ciita | Mm00482914_m1 |
| Clca1 | Mm01320697_m1 |
| Clca2 | Mm00724513_m1 |
| Clca4a | Mm01614382_m1 |
| Clcn2 | Mm00438245_m1 |
| Clcn5 | Mm00443851_m1 |
| Cldn23 | Mm00510971_s1 |
| Cldn3 | Mm00515499_s1 |
| Cldn4 | Mm00515514_s1 |
| Cldn5 | Mm00727012_s1 |
| Cldn8 | Mm00516972_s1 |
| Cox6b2 | Mm01333764_g1 |
| Cxcl1 | Mm04207460_m1 |
| Cxcl15 | Mm00441263_m1 |
| Cyp26b1 | Mm00558507_m1 |
| Cyp2c55 | Mm00472168_m1 |
| Cyp2d26 | Mm00472520_m1 |
| Cyp2d34 | Mm00661938_m1 |
| Cyp3a13 | Mm00484110_m1 |
| Cyp4b1 | Mm00484138_m1 |
| Cyp4f14 | Mm00491623_m1 |
| Defa-rs1 | Mm00655850_m1 |
| Defb1 | Mm00432803_m1 |
| Defb2 | Mm00657074_m1 |
| Defb3 | Mm04214158_s1 |
| Defb8 | Mm00838330_m1 |
| Duox2 | Mm01326247_m1 |
| Duoxa2 | Mm00470560_m1 |
| Dusp3 | Mm00459216_m1 |
| Dusp6 | Mm00518185_m1 |
| Egfr | Mm00433023_m1 |
| Egln3 | Mm00472200_m1 |
| Egr1 | Mm00656724_m1 |
| Ephx1 | Mm00468752_m1 |
| Fcgbp | Mm01175503_m1 |
| Fgf11 | Mm00679875_m1 |
| Fhl1 | Mm04204611_g1 |
| Fos | Mm00487425_m1 |
| Foxp3 | Mm00475162_m1 |
| Fpr2 | Mm00484464_s1 |
| Fpr3 | Mm01962454_s1 |
| Fut2 | Mm00490152_s1 |
| Fut8 | Mm00489795_m1 |
| Gadd45b | Mm00435123_m1 |
| Gadd45g | Mm01352550_g1 |
| Gapdh | Mm99999915_g1 |
| Gata3 | Mm00484683_m1 |
| Gpx1 | Mm00656767_g1 |
| Gpx3 | Mm00492427_m1 |
| Gsta4 | Mm00494803_m1 |
| Gstk1 | Mm00504022_m1 |
| Gstm3 | Mm00833923_m1 |
| Gstm5 | Mm00515890_m1 |
| Gstp1 | Mm04213618_gH |
| Gstt1 | Mm00492506_m1 |
| Gusb | Mm00446953_m1 |
| Hdac7 | Mm00469527_m1 |
| Hif1a | Mm00468869_m1 |
| Hif3a | Mm00469375_m1 |
| Hspa8 | Mm01731394_gH |
| Hspbp1 | Mm00481069_m1 |
| Ifi44 | Mm00505670_m1 |
| Ifng | Mm99999071_m1 |
| Il10 | Mm01288386_m1 |
| Il12a | Mm00434169_m1 |
| Il12b | Mm01288989_m1 |
| Il13 | Mm00434204_m1 |
| Il17d | Mm01313472_m1 |
| Il17f | Mm00521423_m1 |
| Il18 | Mm00434226_m1 |
| Il18bp | Mm00456733_m1 |
| Il1b | Mm01336189_m1 |
| Il1rn | Mm01337566_m1 |
| Il22 | Mm00444241_m1 |
| Il23a | Mm00518984_m1 |
| Il3ra | Mm00434273_m1 |
| Il4ra | Mm00439634_m1 |
| Il6 | Mm00446190_m1 |
| Irf1 | Mm01288580_m1 |
| Irf7 | Mm00516788_m1 |
| Irf8 | Mm00492567_m1 |
| Itga5 | Mm00439797_m1 |
| Itgal | Mm00801807_m1 |
| Jun | Mm00495062_s1 |
| Kit | Mm00445212_m1 |
| Klf5 | Mm00456521_m1 |
| Klrg1 | Mm00516879_m1 |
| Lbp | Mm00493139_m1 |
| Lifr | Mm00442940_m1 |
| Ltb | Mm00434774_g1 |
| Ly86 | Mm00440240_m1 |
| Map2k6 | Mm00803694_m1 |
| Marveld2 | Mm01282909_m1 |
| Mcoln2 | Mm00509849_m1 |
| Mep1a | Mm00484970_m1 |
| Mki67 | Mm01278617_m1 |
| Mrc1 | Mm00485148_m1 |
| Msi1 | Mm01203522_m1 |
| Muc1 | Mm00449604_m1 |
| Muc13 | Mm00495397_m1 |
| Muc2 | Mm01276696_m1 |
| Muc20 | Mm00524818_m1 |
| Muc3 | Mm01207064_m1 |
| Muc4 | Mm00466886_m1 |
| Myc | Mm00487804_m1 |
| Nfe2l2 | Mm00477784_m1 |
| Nfe2l3 | Mm00477788_m1 |
| Nlrp1b | Mm01241387_m1 |
| Nlrp2 | Mm00624616_m1 |
| Nlrp3 | Mm00840904_m1 |
| Nlrp6 | Mm00460229_m1 |
| Nos2 | Mm00440502_m1 |
| Nos3 | Mm00435217_m1 |
| Nox1 | Mm00549170_m1 |
| Noxa1 | Mm00549172_m1 |
| Npas2 | Mm00500848_m1 |
| Nqo1 | Mm01253561_m1 |
| Nqo2 | Mm01332867_m1 |
| Oas2 | Mm00460961_m1 |
| Olfm4 | Mm01320260_m1 |
| Pigr | Mm00465049_m1 |
| Pla2g10 | Mm01344436_g1 |
| Pla2g12a | Mm00458226_m1 |
| Pla2g2a | Mm00448160_m1 |
| Pla2g3 | Mm01191142_m1 |
| Pla2g4c | Mm01195718_m1 |
| Pnpt1 | Mm00466286_m1 |
| Ppif | Mm00506384_m1 |
| Prf1 | Mm00812512_m1 |
| Ptgs1 | Mm00477214_m1 |
| Ptgs2 | Mm00478374_m1 |
| Rcan1 | Mm00627762_m1 |
| Reg3b | Mm00440616_g1 |
| Reg3g | Mm00441127_m1 |
| Reg4 | Mm00471115_m1 |
| Retnlb | Mm00445845_m1 |
| Rorc | Mm01261022_m1 |
| Rplp1 | Mm02601846_g1 |
| S100a8 | Mm01220132_g1 |
| S100a9 | Mm00656925_m1 |
| Saa3 | Mm00441203_m1 |
| Scnn1a | Mm00803386_m1 |
| Selm | Mm00459806_m1 |
| Sgms1 | Mm00522643_m1 |
| Sgms2 | Mm00512327_m1 |
| Slc10a3 | Mm04210162_g1 |
| Slc12a2 | Mm01265951_m1 |
| Slc15a1 | Mm04209483_m1 |
| Slc19a1 | Mm00446220_m1 |
| Slc22a1 | Mm00456303_m1 |
| Slc22a4 | Mm00457739_m1 |
| Slc26a3 | Mm00445313_m1 |
| Slc4a2 | Mm00436617_m1 |
| Slc9a2 | Mm01237129_m1 |
| Slpi | Mm00441530_g1 |
| Sod2 | Mm01313000_m1 |
| Sod3 | Mm01213380_s1 |
| Sult1a1 | Mm00467072_m1 |
| Sult1b1 | Mm01213937_m1 |
| Tbp | Mm00446973_m1 |
| Tbx21 | Mm00450960_m1 |
| Tff3 | Mm00495590_m1 |
| Thpo | Mm00437040_m1 |
| Tlr12 | Mm01180204_s1 |
| Tlr4 | Mm00445273_m1 |
| Tlr5 | Mm00546288_s1 |
| Tlr7 | Mm00446590_m1 |
| Tlr9 | Mm00446193_m1 |
| Tnf | Mm00443258_m1 |
| Tnfrsf4 | Mm00442039_m1 |
| Tnfsf13 | Mm03809849_s1 |
| Tnfsf18 | Mm00839222_m1 |
| Tslp | Mm01157588_m1 |
| Txnip | Mm01265659_g1 |
| Txnrd1 | Mm00443675_m1 |
| Ubc | Mm01201237_m1 |
| Wnt11 | Mm00437327_g1 |
| Wnt16 | Mm00446420_m1 |
| Wnt2b | Mm00437330_m1 |
| Wnt5a | Mm00437347_m1 |
| Wnt5b | Mm01183986_m1 |
| Xbp1 | Mm00457357_m1 |
